# Towards a better detection of patients at-risk of linezolid toxicity in clinical practice: a prospective study in three Belgian hospital centers

**DOI:** 10.3389/fphar.2024.1310309

**Published:** 2024-01-19

**Authors:** Hélène Thirot, David Fage, Antonia Leonhardt, Philippe Clevenbergh, Tatiana Besse-Hammer, Jean Cyr Yombi, Olivier Cornu, Caroline Briquet, Maya Hites, Frédérique Jacobs, Gert-Jan Wijnant, Sebastian G. Wicha, Frédéric Cotton, Paul M. Tulkens, Anne Spinewine, Françoise Van Bambeke

**Affiliations:** ^1^ Pharmacologie cellulaire et Moléculaire, Louvain Drug Research Institute, Université catholique de Louvain, Brussels, Belgium; ^2^ Clinical Pharmacy, Louvain Drug Research Institute, Université catholique de Louvain, Brussels, Belgium; ^3^ Department of Clinical Chemistry, Laboratoire hospitalier universitaire de Bruxelles (LHUB-ULB), Brussels, Belgium; ^4^ Department of Clinical Pharmacy, Institute of Pharmacy, University of Hamburg, Hambourg, Germany; ^5^ University Hospital Brugmann, Université libre de Bruxelles, Brussels, Belgium; ^6^ Cliniques universitaires Saint-Luc, Université catholique de Louvain, Brussels, Belgium; ^7^ Hôpitaux universitaires de Bruxelles-Erasme (HUB), Université libre de Bruxelles, Brussels, Belgium

**Keywords:** linezolid, thrombocytopenia, adverse drug reaction, therapeutic drug monitoring, anemia, Buzelé score, neurotoxicity

## Abstract

**Introduction:** Linezolid is a last-resort antibiotic for infections caused by multidrug-resistant microorganisms. It is widely used for off-label indications and for longer than recommended treatment durations, exposing patients at higher risk of adverse drug reactions (ADRs), notably thrombocytopenia. This study aimed to investigate ADR incidence and risk factors, identify thrombocytopenia-related trough levels based on treatment duration, and evaluate the performance of predictive scores for ADR development.

**Methods:** Adult in- and outpatients undergoing linezolid therapy were enrolled in three hospitals and ADRs and linezolid trough levels prospectively monitored over time. A population pharmacokinetic (pop-PK model) was used to estimate trough levels for blood samples collected at varying times.

**Results:** A multivariate analysis based on 63 treatments identified treatment duration ≥10 days and trough levels >8 mg/L as independent risk factors of developing thrombocytopenia, with high trough values correlated with impaired renal function. Five patients treated for >28 days did not develop thrombocytopenia but maintained trough values in the target range (<8 mg/L). The Buzelé predictive score, which combines an age-adjusted Charlson comorbidity index with treatment duration, demonstrated 77% specificity and 67% sensitivity to predict the risk of ADR.

**Conclusion:** Our work supports the necessity of establishing guidelines for dose adjustment in patients with renal insufficiency and the systematic use of TDM in patients at-risk in order to keep trough values ≤8 mg/L. The Buzelé predictive score (if ≥7) may help to detect these at-risk patients, and pop-PK models can estimate trough levels based on plasma samples collected at varying times, reducing the logistical burden of TDM in clinical practice.

## Introduction

Linezolid is an oxazolidinone antibiotic with excellent oral bioavailability, high tissue penetration and potent activity against drug-resistant Gram-positive bacteria such as methicillin-resistant *Staphylococcus aureus* (MRSA), methicillin-resistant *Staphylococcus epidermidis* (MRSE) and vancomycin-resistant Enterococci (VRE). In Europe, linezolid is indicated for the treatment of community-acquired and nosocomial pneumonia, complicated and uncomplicated skin and skin structure infections (SSSI), at a daily dose of 600 mg every 12 h, according to the European Summary of Product Characteristics (SmPC) as well as for the treatment of vancomycin-resistant *Enterococcus faecium* infections and uncomplicated SSSI (400 mg every 12 h) in the US label (Zyvox US perscribing information 2023). However, we previously showed that this drug is also widely prescribed for a number of off-label indications in the Belgian practice, including bone and joint infections (BJIs), urinary tract infections (UTIs), endocarditis and bacteremia (Thirot, Briquet et al., 2021). Some of these infections, in particular BJIs, typically require longer treatment durations than the currently recommended maximum of 28 days, raising concerns for long-term patient safety during linezolid treatment.

The most serious adverse drug reactions (ADR) reported in the SmPC or US label include hematological disorders (anemia and thrombocytopenia), peripheral and optic neuropathy, or lactic acidosis, with a respective prevalence of 1%–10%, 0.1%–1%, and 0.01%–0.1% in the last update of the SmPC (Zyvoxid Belgian SmPC, 2023). Yet, higher prevalence is observed in clinical practice for thrombocytopenia (15%–55%) (Niwa, Suzuki et al., 2009; Ichie, Suzuki et al., 2015; Thirot, Briquet et al., 2021; Inoue, Takekuma et al., 2023) and anemia (10%–20%) (Hanai, Matsuo et al., 2016; Dai, Jiang et al., 2021; Qin, Liu et al., 2021), while the others ADR remain rarely reported (Mao, Dai et al., 2018; Brandariz-Núñez, Hernández-Corredoira et al., 2019; Lan, Ahmad et al., 2020). Known risk factors for hematological toxicity include impaired renal function, long treatment duration, presence of comorbidities (evaluated by the Charlson index, a score aiming at predicting the risk of death based on the number and the severity of comorbidities) and low basal platelet count (Charlson, Pompei et al., 1987; Buzele, Lemaignen et al., 2015; Ichie, Suzuki et al., 2015; Hanai, Matsuo et al., 2016; Choi, Lee et al., 2019; Lima, Brito et al., 2020; Dai, Jiang et al., 2021; Thirot, Briquet et al., 2021; Inoue, Takekuma et al., 2023). Two scores have been designed to predict the risk of developing ADRs with linezolid: the Buzelé’s score includes an age-adjusted Charlson comorbidity index and treatment duration (Buzele, Lemaignen et al., 2015) and the Gonzalez-Del Castillo’s score (specific for hematological toxicity) includes liver or cerebrovascular diseases, baseline platelet count and glomerular filtration rate (Gonzalez-Del Castillo, Candel et al., 2017). Yet, these scores are not applied in clinical practice.

To minimize the risk for the development of hematological side effects during linezolid treatment, weekly monitoring of blood cell counts is recommended. Furthermore, considering the relatively high interpatient pharmacokinetic (PK) variability of linezolid, therapeutic drug monitoring (TDM) of drug concentrations in blood has been proposed to further improve treatment efficacy and safety (Pea, Cojutti et al., 2017; Cojutti, Merelli et al., 2019). The therapeutic target for linezolid is to achieve an area under the concentration-time curve over 24 h divided by the minimal inhibitory concentration (AUC/MIC) > 80–100 h^−1^ corresponding to a linezolid trough concentration (C_min_) within a therapeutic range of 2–8 mg/L (Lin, Hu et al., 2022). Conversely, supratherapeutic C_min_ ≥ 9 mg/L have been linked to an increased risk for thrombocytopenia (Cattaneo, Gervasoni et al., 2016).

Although recommended in some local guidelines (Lin, Hu et al., 2022), linezolid monitoring is not yet widely implemented in clinical practice, including in Belgium, where it is used in only a few hospitals, to evaluate efficacy, but not to assess (long-term) toxicity risk. Therefore, the objectives of this study were i) to investigate the incidence and risk factors of ADRs in patients treated with linezolid in clinical practice, ii) to identify trough levels associated with (hematological) toxicity in function of treatment duration and iii) to evaluate the performance of two published predictive scores for the development of ADR.

## Methods

This prospective multicentric study was performed in three Belgian university hospitals (*Cliniques universitaires Saint Luc*—945 beds, *Centre hospitalier universitaire Brugmann*—854 beds, *Cliniques universitaires de Bruxelles, Erasme*—1,048 beds, respectively) between May 2021 and March 2023. This study was approved by the principal and local ethic committees and registered on clinicaltrialregister.eu (EudraCT number: 2020-005772-35).

### Patients

In Belgium, linezolid is exclusively prescribed by authorized doctors in hospitals (infectious diseases specialists), and dispensed to outpatients by the hospital pharmacy. Hospitalized and/or ambulatory adult (≥18 years) patients treated with linezolid (600 mg q12h orally or intravenously) for a period of at least 3 days (to achieve steady-state concentrations) were included after signing an informed consent form. Patients with baseline hematological disorders (defined as hemoglobin level <8 g/dL and/or platelet counts <75 10^9^/L) were excluded.

### Sample size calculation

The required sample size for this clinical study was estimated based on a statistical power calculation (using a two-sided two-sample equal-variance t-test) and on the results of a previous study (Dong, Xie et al., 2014). Power calculations indicated the need for a sample size of at least 20 patients per group (thrombocytopenia vs. no thrombocytopenia) to be able to observe a statically significant difference in linezolid C_min_ in patients with and without hematotoxicity (see Supplementary Table S1 for more details).

### Data collection

Data were collected from the first to the last day of treatment. If ADRs occurred during the treatment, patients were followed during one-month post-treatment to assess recovery. Depending on the status of the patient (in- or outpatient) data were extracted from hospitalization reports, electronic medical records (EMR), visit reports (in general every 15 days) and a weekly phone call for outpatients. The following data were extracted from the EMR: demographic data, comorbidities [to calculate the Charlson comorbidity index (Charlson, Pompei et al., 1987)], body weight, renal function [creatinine level and glomerular filtration rate estimated with CKD-EPI (Chronic Kidney Disease Epidemiology Collaboration) formula (Levey, Stevens et al., 2009)], hemogram (performed concomitantly to linezolid TDM in most of the cases and including hemoglobin concentration, platelets, neutrophils, white blood cell counts), type of infection, microbiological data, previous antibiotic treatments, reason for linezolid prescription, dosage, treatment duration, route of administration, any change during linezolid treatment (change of dosage or early stop based on TDM, toxicity or any other reason), and comedications that could lead to drug-drug interaction with linezolid (increasing the risk of serotonin syndrome or thrombocytopenia or modifying linezolid pharmacokinetics) (Pea, Furlanut et al., 2010; Rondina, Walker et al., 2010; Buckley, Dawson et al., 2014).

### ADRs definition and risk scores

If ADRs occurred during the treatment, the following data were collected: ADR description, time of onset, management, recovery, potential alternative cause. Thrombocytopenia was defined as a platelet count <150 10^9^/L and a reduction of 30% from baseline (Erkurt, Kaya et al., 2012; Hanai, Matsuo et al., 2016). Anemia was defined as a hemoglobin value <12 g/dL and a reduction of 30% from baseline (Cappellini and Motta, 2015; Hanai, Matsuo et al., 2016). The potential association between linezolid usage and the observed ADR was calculated using the Naranjo adverse drug reaction probability scale (Naranjo, Busto et al., 1981). Two predictive scores for linezolid toxicity development were applied to the whole population to verify their applicability. The first refers to the prediction of ADRs with linezolid (Buzele, Lemaignen et al., 2015). It includes an age-adjusted Charlson comorbidity index, and the treatment duration. A score ≥7 is proposed as associated with ADRs. The second concerns the prediction of linezolid hematological toxicity through a score including the presence of a moderate to severe liver disease, cerebrovascular disease, a baseline platelet count <90 10^9^/L and a baseline glomerular filtration rate (GFR) < 50 mL/min (Gonzalez-Del Castillo, Candel et al., 2017). A score between 0 and 4 represents a low risk, between 5 and 10 an intermediate risk and >10, a high risk of developing hematological toxicity.

### Linezolid therapeutic monitoring

Linezolid monitoring was performed every 7 days on average for hospitalized patients and the day of the follow-up consultation for ambulatory patients (on average every 15 days for treatments longer than 28 days). As the administered linezolid dose was 600 mg q12h, venous blood samples were collected at the 12 h time point right before administration of the next dose. In case the 12 h sampling time could not be respected (i.e., sampling >2 h before or after the target time for ambulatory patients taking linezolid orally), the trough values at 12 h were simulated based on the actual measured sample values using an established population pharmacokinetic model for linezolid (Plock, Buerger et al., 2007). This pharmacometric approach allowed comparison of all patient trough values at 12 h despite varying sampling times.

### Linezolid sample preparation and analysis

Blood samples were collected without any anticoagulant and centrifuged for 10 min at 1968 *g* and sera were frozen at −80°C until analysis. Linezolid quantification was performed according to the published method (Fage, Deprez et al., 2021). Briefly, the extraction and the sample deproteinization were done with organic solvent. After centrifugation, the supernatant was recovered, evaporated and the residue containing linezolid was reconstituted with 200 μL of 2% acetonitrile and 0.6% phosphate solution at pH 5. The mix was then injected onto an ultra-performance liquid chromatography system coupled to a photodiode array detector (UPLC-PDA). The assay was linear in the 0.75–50.00 mg/L range.

### Pharmacokinetic modelling

A literature research was performed to identify available oral linezolid pop-PK models derived from patient populations comparable to the one in this study, excluding those using covariates that were not assessed in our patients. The predictive performance and fit of five eligible models (Plock, Buerger et al., 2007; Abe, Chiba et al., 2009; Boak, Rayner et al., 2014; Matsumoto, Shigemi et al., 2014; Tsuji, Holford et al., 2017) was evaluated and Bland-Altman-plots were used to compare agreement between the model results (see Methods and Results in Supplementary Material). The Plock et al. model (Plock, Buerger et al., 2007) was selected to simulate trough concentrations at 12 h, as the studied population was the most similar to our patients and it showed the best fit with the data observed in our study. All simulations were performed in NONMEM (version 7.4.3, ICON, Gaithersburg, MD, United States).

### Statistical analysis

Statistical analyses were performed using IBM SPSS statistics version 26.0 (IBM, Armonk, NY) or Graphpad prism version 9.5.1 (GraphPad Software, San Diego, CA). Distribution and normality of quantitative data were analyzed with Shapiro-Wilk test (*n* < 100). As data distribution was predominantly not normal, data are shown as median and range. Continuous variables were analyzed with Mann-Whitney U test. Pearson’s Chi-squared test or Fisher exact test was used to compare categorical variables (according to the conditions of each test). Correlation between two parameters were assessed with Spearman correlation test and presented with the Spearman correlation coefficient r_s_ and correlation was significant when *p*-value < 0.05. Correlation was positive with a positive r_s_ and negative with a negative r_s_. Scores sensitivity was calculated with the formula true positive divided by the observed patients with ADR. Specificity was calculated by the division of true negatives divided by the total without ADR. Time of onset of ADR were assessed with a Kaplan-Meier survival curve. Identification of risk factors for hematological disorders was performed through a univariate analysis and multivariate logistic regression. Variables with a *p*-value < 0.1 were included in the final multivariate model (instead of 0.2 based on the small number of patients and high number of variables) but had to be limited in view of the small sample size. This was notably performed by testing the collinearity between variables using the variance inflation factor (VIF): when VIF was >2, the corresponding parameter was removed from the analysis and not included in the multivariate analysis. A backward procedure was used to select final interesting variables. The goodness-of-fit of the final model was evaluated with the Hosmer-Lemeshow test. Odds ratio (OR) with 95% confidence interval was used to present the results of the final model. ROC curves, a widely used method to figure out the accuracy of a diagnostic test and establish a cut-off value for the diagnosis of the studied disease, were used to draw plots of sensitivity (true positive rate) by 1-specificity (false positive rate) at every test value, by dichotomizing patients into having the disease (here thrombocytopenia) or not. The cut-off point was determined as the value where the sensitivity and specificity are highest (Hajian-Tilaki, 2013).

## Results

### Study subjects

In total, 59 patients met the inclusion criteria and were included in the study, representing 63 treatments (3 patients received 2 or 3 linezolid treatments during the study period). Patient baseline characteristics are described in Table 1. On average, the patients were old (median age = 65 years), slightly overweight (BMI = 28.1 kg/m^2^), and presented with mild renal insufficiency (median GFR = 69 mL/min/1.73 m^2^). Median of GFR was significantly lower among inpatients (62 mL/min/1.73 m^2^) than outpatients (82 mL/min/1.73 m^2^) [*p*-value, 0.037]. The majority of patients received their treatment at the hospital (76.2%), with a minority in the intensive care unit (12.5% of inpatients).

**TABLE 1:** Patient baseline characteristics (63 patients).

Patients' characteristics	*N (%) or median (range)*
Male/female	39/24 (61.9/38.1)
Age (years)	65 (31–97)
Weight (kg)	80 (40–154)
Body mass index (kg/m^2^)	28.1 (14.9–46)
Glomerular filtration rate (mL/min/1.73 m^2^)	69 (5–134)
Creatinine level (mg/dL)	1 (0.32–8.16)
Charlson index	2 (0–6)
Inpatients/outpatients	48/15 (76.2%/23.8%)
Intensive care unit (ICU)/general ward	6/42 (12.5%/87.5%)

### Linezolid treatment

Linezolid was administered as 600 mg twice daily via the oral (*n* = 54/63) or intravenous (IV, *n* = 9/63, with one switch from IV to oral) routes. It was prescribed as a first line in 12 patients (19%) or second line after vancomycin in 21 patients (33.3%), or after another antibiotic in 30 patients (47.7%).

The reasons for selecting linezolid were: oral availability [switch to an oral therapy (14.3%) or only oral option (7.9%)], efficacy [susceptibility profile of the isolated strain (49.2% including 4.8% of VRE) or no effect of the first prescribed antimicrobial (4.8%)], safety reasons [renal insufficiency (6.3%), allergies to other drugs (3.2%), or ADRs of the first prescribed antimicrobial (4.8%)] or not specified (9.6%).

The type of infections treated with linezolid and the corresponding median treatment duration are summarized in Table 2. A majority of patients were treated for BJI, SSSI, secondary bacteremia or endocarditis. Treatment durations for the different types of infections were in the limit recommended in the SmPC (≤28 days), but longer for 10/22 (45%) patients with BJIs (range = 9–121 days). These infections were mainly caused by enterococci (25.4% *E. faecium* and 4.8% vancomycin-resistant enterococci), *methicillin-resistant Staphylococcus epidermidis* (20.6%), *methicillin-resistant Staphylococcus aureus* (19%).

**TABLE 2:** Indication and duration of linezolid treatment.

Infection	N (%)	Median treatment duration (range)
BJI	22 (34.9)	28 (9–121)
SSSI	10 (15.9)	11 (8–17)
Secondary bacteremia	9 (14.3)	10 (5–23)
Endocarditis	6 (9.5)	13.5 (9–19)
Septic shock	5 (7.9)	8 (6–10)
UTI	2 (3.2)	6 (5–7)
Pneumonia	3 (4.8)	7 (7–10)
others	6 (9.5)	nd^a^

Acronyms: BJI, bone and joint infection; SSSI, skin and soft tissue infection; UTI, urinary tract infection.

^a^ nd: not determined as the type of infections included in this category are different.

### Linezolid TDM

**FIGURE 1:**
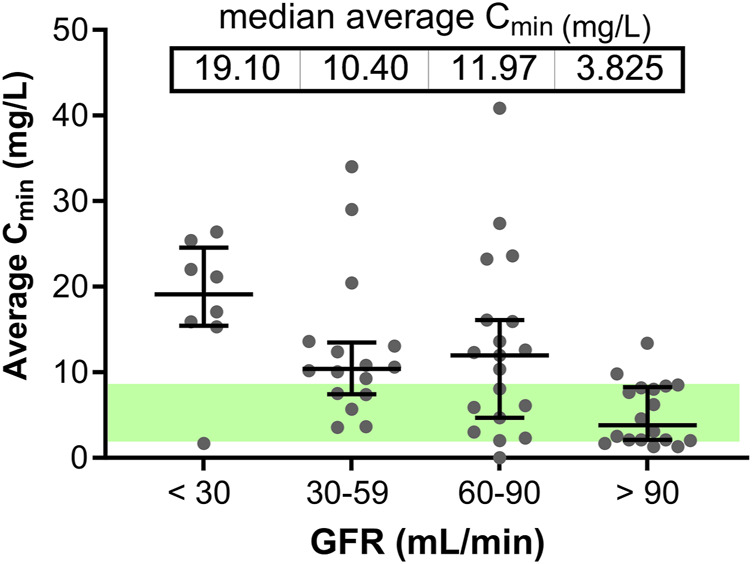
C_min_ in relation to the renal function of individual patients. Patients are categorized as showing a severe, moderate, mild renal insufficiency, or a normal renal function based on their GFR values. Grey dots correspond to the average C_min_ for each individual patient; the horizontal lines show the median and interquartile range. The green zone highlights the target C_min_ window (2–8 mg/L).

Linezolid concentrations were measured in 120 blood samples. On average, 2 samples were collected per patient (range 1–6 depending on treatment duration). 16/120 samples were not collected at the target 12 h of sampling (C_min_) due to practical reasons; in these cases, the C_min_ at 12 h was estimated based on simulations using the population-PK model described by Plock et al. (Plock, Buerger et al., 2007) and included in the final calculations. High interpatient PK variability for linezolid was observed, with C_min_ values ranging from the lower limit of quantification to 46.4 mg/L for the same 600 mg dose (Supplementary Figure S1). Renal function was identified as the main factor affecting C_min_ (Supplementary Figures S1, S2; Supplementary Table S2). Patients with C_min_ > 8 mg/L had a lower GFR (median value: 58.5 mL/min/1.73 m^2^) than those with C_min_ ≤ 8 mg/L (median value: 96 mL/min/1.73 m^2^; *p* = 0.004 for this difference), so that patients with mild to severe renal insufficiency were at higher risk of overdosing (Figure 1). Age was also positively correlated with C_min_ (r_s_ = 0.5509; *p*-value <0.0001), although this association is likely the result of the confounding effect of age on GFR (negative correlation, r_s_ = −0.6123; *p*-value < 0.0001).

Subgroup analyses were performed in order to identify specific subpopulations that may be at higher risk of under- or overexposure (Supplementary Table S2). Obese patients (*n =* 24) showed a median C_min_ of 8.3 mg/L, which is actually at the upper limit of the target interval. As expected, patients with GFR >100 mL/min/1.73 m^2^ (*n =* 14) had significantly lower C_min_ than those with GFR <100 mL/min/1.73 m^2^ (median C_min_ value: 3.8 mg/L vs. 10.8 mg/L). Importantly, all 6 ICU patients had low C_min_ values (0–5.7 mg/L). Conversely, patients with hepatic disorders (*n =* 8) tended to have elevated C_min_ (median value: 8.7 mg/L). Of note, among 4 patients with GFR between 60 and 90 mL/min/1.73 m^2^ and C_min_ > 20 mg/L, two had hepatic disorders (Supplementary Figures S1, S2).

Overall, only 38.3% (46/120) C_min_ values were within the recommended 2–8 mg/L therapeutic range, with 48.3% (58/120) values >8 mg/L (supratherapeutic) and 13.3% (16/120) values <2 mg/L (subtherapeutic). Correcting for the number of samples per patient, these results indicate that more than half of the patients (35/63) were exposed to supratherapeutic concentrations while 5 were underdosed. Linezolid dose adaptation based on TDM results was applied in 5 patients with C_min_ > 8 mg/L (reduction from 600 mg twice to once daily) and in 1 patient with C_min_ < 2 mg/L (increase from 600 mg twice to trice daily) (Supplementary Figure S3). Dose reduction brought C_min_ back in the target values for 4/5 patients but did not prevent thrombocytopenia in 2 of them, either because platelets were already decreasing before dose readjustment (patient B.15) or because they were already low from day 1 (patient B.26). Dose increase in patient B.10 brought C_min_ back into the desired range.

### Adverse drug reactions

**FIGURE 2:**
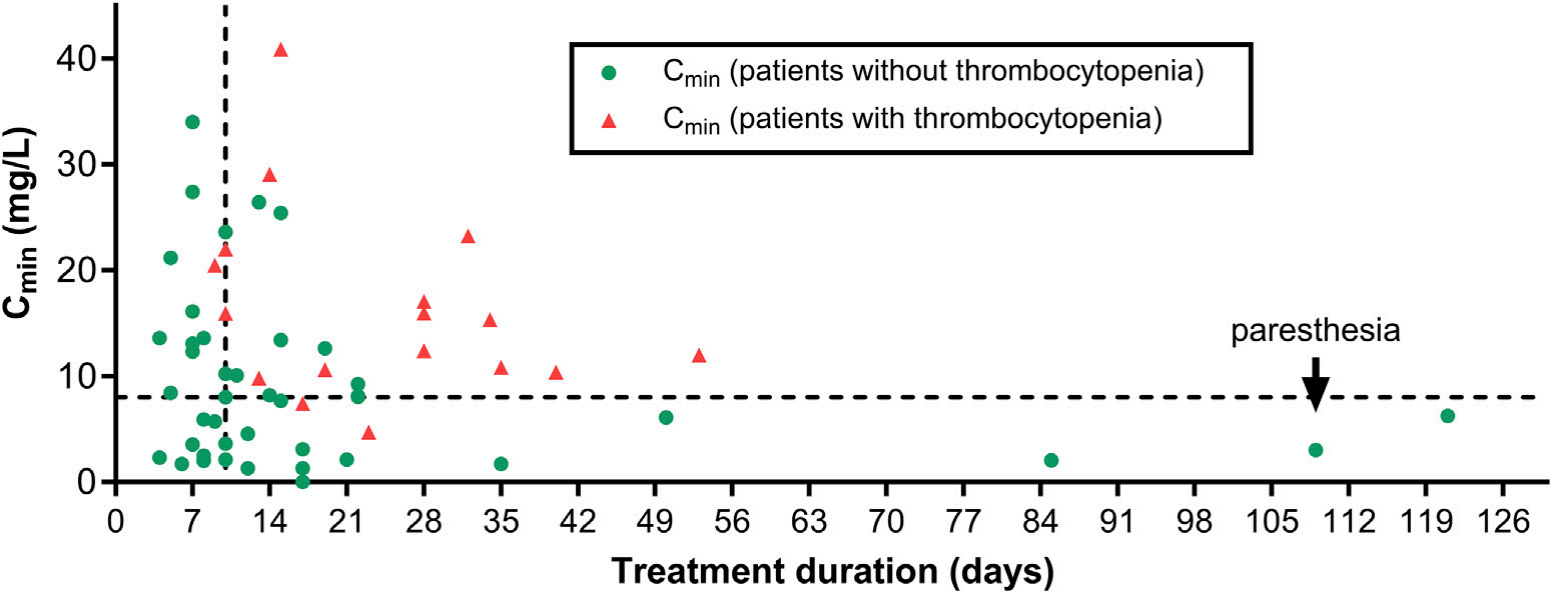
Average C_min_ value for each patient vs. individual treatment duration. Symbols distinguish patients who did not (green dots) or did (red triangles) develop thrombocytopenia. The horizontal dotted line corresponds to the maximal target C_min_ (8 mg/L) and the vertical dotted line, to a treatment duration of 10 days. The black arrow points to the patient who developed paresthesia.

ADRs occurred in 52.4% of linezolid treatments, with up to 4 ADRs per patient. These ADRs and their corresponding Naranjo probability score are shown in Table 3. According to this score, the majority of ADRs were probably associated with linezolid. Hematological disorders were detected in 20 patients, among whom 11 developed thrombocytopenia; 2 anemia; and 7 both anemia and thrombocytopenia. Thrombocytopenia and anemia appeared after a median of 13 days and 18 days, respectively. Five patients had a thrombocytopenia of grade 2 (50–75 10^9^ platelets/L) when their treatment was stopped. Among patients developing anemia, 6 received a blood transfusion, and among those with thrombocytopenia, 2 received a platelet transfusion. Importantly, among the 20 patients with hematological toxicity, the median trough value was 12.4 mg/L and 15 showed trough values above the recommended therapeutic range (2–8 mg/L, Figure 2). Patients developing thrombocytopenia had a treatment duration ≥9 days and the majority (15/17 with C_min_ values) showed supratherapeutic linezolid levels (>8 mg/L).

**TABLE 3:** Adverse drug reactions observed during linezolid treatment and their Naranjo score.

ADRs	N (% in 63 treatments)	Naranjo score Median (range)^a^	Day of onset Median (range)
Thrombocytopenia	18 (28.1)	5 (4–7)	13 (7–28)
Anemia	10 (15.6)	7 (4–8)	19 (3–35)
Metallic taste	9 (14.1)	7 (5–9)	7 (2–17)
Gastro intestinal disorders	18 (28.1)	4 (3–6)	6 (3–50)
Tinnitus	1 (1.6)	6	2
Mycosis	2 (3.2)	6.5 (6–7)	7
Paresthesia	1 (1.6)	4	108
Hepatic disorders	2 (3.2)	5.5 (4–7)	10.5 (4–17)

^a^ Score 1 to 4 = Possibly associated; Score 5 to 8 = Probably associated; Score ≥9 = Definitely associated.

Gastrointestinal disorders were noticed in 18 patients and included diarrhea, nausea, vomiting, or loss of appetite. Metallic taste occurred in 9 patients and appeared between 9 and 17 days. Six patients experiencing metallic taste also developed thrombocytopenia, with metallic taste reported earlier in 5/6 patients. Higher C_min_ (median >8 mg/L) were observed in patients developing thrombocytopenia, anemia or metallic taste, but the difference with patients without ADR reached significance only for thrombocytopenia; Supplementary Figure S4).

No serotonin syndrome was observed during the study, despite the mention of comedications that can increase the risk in patients’ files (Supplementary Table S3). Twenty-five patients took at least 1 serotonergic agent; 10 patients, 2 serotonergic agents; and 1 patient, 3 serotonergic agents. Antidepressants were prescribed in 16 patients (7 patients with 2 antidepressants).

Eleven patients had to stop their linezolid treatment because of toxicity, among whom, 10 for hematological toxicity (after 9–34 days of treatment) and one, for paresthesia after 109 days. In this last patient, the trough value was 4.9 mg/L when the treatment was stopped (Figure 2).

### Predictive scores for the development of ADRs

Supplementary Tables S4, S5 shows individual Gonzalez-Del Castillo and Buzelé scores for patients developing hematological toxicity or any ADR. The predictive score for hematological toxicity developed by Gonzalez-Del Castillo et al. (Gonzalez-Del Castillo, Candel et al., 2017) did not prove useful to predict the risk of adverse reaction in our population, as all patients had a score <5 (Table 4). Conversely, the Buzelé et al. score (Buzele, Lemaignen et al., 2015) was ≥7 and significantly higher for patients developing any type of ADR [*p*-value, 0.001] (Table 4). The specificity and sensitivity of the Buzelé test for the detection of ADR are 76.7% (95% CI = 59.7–89.2%) and 66.6% (95% CI = 49.8–81.1%), respectively (Supplementary Figure S5).

**TABLE 4:** Contingency table for predictive scores.

Gonzalez-Del Castillo score				Buzelé score			
	Score ≤4	Score ≥5	Total		Score <7	Score ≥7	Total
No HT^a^	43	0	43	No ADR	23	7	30
HT	20	0	20	ADR	11	22	33
Total	63	0	63	Total	34	29	63

^a^ HT, hematological toxicity.

### Risk factors for developing thrombocytopenia

As thrombocytopenia was the most frequent ADR in our population, our further analyses focused on this ADR. A significant difference among patients with (*n* = 18) and without (*n* = 45) thrombocytopenia was observed for the following parameters (Supplementary Table S6): median linezolid C_min_ (15.3 mg/L vs. 7.6 mg/L), treatment duration (21 days vs. 10 days), GFR (50 mL/min/1.73 m^2^ vs. 74 mL/min/1.73 m^2^), basal platelet count (253 vs. 338 10^9^/L), and previous vancomycin treatment (55.6% vs. 24.4% of patients). A positive correlation was found between the decrease in platelet count during treatment compared to baseline levels and average trough concentrations (r_s_ = 0.3642; *p*-value = 0.0046) (Supplementary Figure S2). Interestingly, while the risk of thrombocytopenia increased as treatment duration was extended, 3 patients completed >80 days of linezolid therapy without signs of hematological toxicity. Of note, their trough values remained within the 2–8 mg/L therapeutic range (Figure 2).

Table 5 shows the results of the univariate and multivariate analysis for the development of thrombocytopenia (see Supplementary Figure S6 for the establishment of cut-ff values to <150 10^9^/L for basal platelet count, <60 mL/min/1.73 m^2^ for GFR [similar cut-off value if using absolute GFR; see Supplementary Figure S7], ≥10 days for treatment duration, and >8 mg/L for C_min_). Based on the results of the univariate analysis, we decided to exclude outpatients from the multivariate analysis as this parameter showed a VIF >2 and is generally associated with a longer treatment duration (median treatment duration in hospitalized patient = 10 days vs. 32 days in ambulatory patients). We also excluded age, which was negatively correlated with renal function. In the resulting multivariate analysis, factors significantly associated with thrombocytopenia were treatment duration longer or equal to 10 days and C_min_ > 8 mg/L.

**TABLE 5:** Univariate and multivariate analysis of parameters associated with the development of thrombocytopenia^a^.

Parameters	OR [CI 95%]	*p*-value	Multivariate analysis adjusted OR [95%]	*p*-value
Age >60 years	3.333 [0.842–13.191]	0.086		
Outpatients	2.943 [0.871–9.943]	0.082		
Basal platelet count <150 10^9^/L	2.588 [0.153–43.760]	0.51		
Previous vancomycin treatment	3.864 [1.221–12.224]	0.021		
GFR <60 mL/min/1.73 m^2^	3.48 [1.114–10.864]	0.032		
Treatment ≥10 days	5.846 [1.199–28.512]	0.029	13.985 [1.596–122.582]	0.017
C_min_ > 8 mg/L	8.214 [1.676–40.264]	0.009	10.07 [1.926–52.663]	0.06

^a^ See Supplementary Figure S6 for the establishment of these cut-off values. Note that ROC curve rather suggests to use a cut-off value of 300 10^9^/L for platelet counts, but this threshold was considered as not clinically-relevant, because it would include the vast majority of the patients and therefore not be discriminant.

### Predictive value of TDM data

**FIGURE 3:**
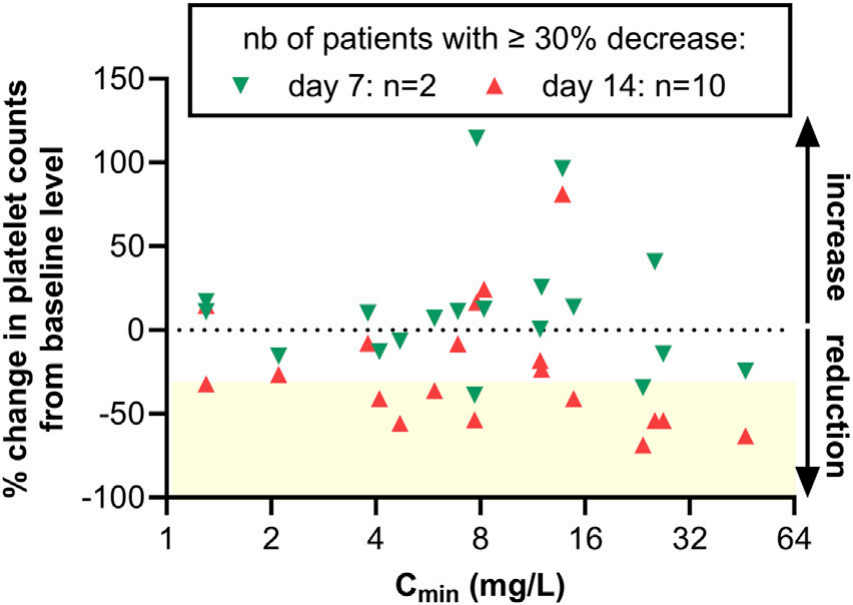
Percentage of change in platelet counts at day 7 vs. day 0 or day 14 vs. day 0. A negative value denotes a decrease in platelet counts over time. The horizontal dashed line corresponds to 0% change, and the yellow box, to a reduction of at least 30%. Green upside triangles: values at day 7; downside triangles: values at day 14. A reduction of at least 30% was seen in 2 patients at day 7 and 10 patients at day 14 out of the 19 patients for which platelets counts were available at both time points.

Although this study was not designed to perform systematic dose readjustment based on TDM data, we exploited the data from patients with a sample collected for platelet counts 7 days after the determination of linezolid C_min_ (at day 7 or 10) in order the determine whether elevated C_min_ values could predict the risk of developing thrombocytopenia in the forthcoming days. Figure 3 shows the change in platelets counts from the pretreatment value (day 0) at day 7 (day at which C_min_ was measured) or 1 week later (day 14). In most of the cases, platelet counts at day 7 were close to the values at day 0 with only 2/19 patients showing >30% decrease in platelet counts. Contrarily, 1 week later, 10/19 patients showed >30% decrease in platelet counts, among whom 5 with C_min_ > 8 mg/L. Seven out of these 10 patients had also platelets counts <150 10^9^/L (not shown), corresponding to the definition of thrombocytopenia adopted in this study.

Supplementary Figure S8 shows the evolution over time of platelets counts in patients for whom at least 2 TDM values were available (panel A) or having developed thrombocytopenia (panel B). Although these data are fragmental, they seem to confirm that elevated C_min_ may precede platelet count reduction. In four patients where linezolid was stopped after 7–14 days and experiencing high C_min_ without thrombocytopenia, platelet numbers continued to decrease over the next week, suggesting the slow reversibility of the process in these patients (Supplementary Figure S9).

## Discussion

Thrombocytopenia used to be considered an uncommon ADR during linezolid treatment occurring in 0.1%–1% of the patients, according to the original release of the SmPC (Thirot, Briquet et al., 2021), which was based on data from registration clinical trials (Rubinstein, Isturiz et al., 2003). While the current European SmPC reports a higher incidence of 1%–10% (Zyvox SmPC, 2018), we observe here thrombocytopenia in 28.6% (18/63) of the treatments, with a Naranjo score of 5-8 indicating it was probably a direct result of linezolid therapy (Naranjo, Busto et al., 1981). This high incidence is comparable to that reported in other post-marketing studies (see, e.g., Niwa, Suzuki et al., 2009; Ichie, Suzuki et al., 2015; Inoue, Takekuma et al., 2023). Anemia and metallic taste, described as frequent ADRs in the last release of the SmPC (Zyvox SmPC, 2018), also emerged as frequent ADRs in our cohort, and were classified as probably associated to the drug.

The multivariate analysis identified long treatment duration (≥10 days) and elevated C_min_ (>8 mg/L) as independent risk factors for thrombocytopenia. Previous vancomycin treatment, age, and impaired renal function (GFR <60 mL/min/1.73 m^2^), also identified in the univariate analysis, are the most probable causes of overdosing, since vancomycin is nephrotoxic, and age is associated with progressive reduction of renal function. These findings are consistent with earlier studies, although threshold values might slightly vary (10–14 days of treatment (Takahashi, Takesue et al., 2011; Chen, Guo et al., 2012; Hirano, Sakamoto et al., 2014), GFR <30–60 mL/min or creatinine clearance <50 mL/min (Takahashi, Takesue et al., 2011; Chen, Guo et al., 2012; Hirano, Sakamoto et al., 2014; Liu, Aoki et al., 2022), C_min_ < 12–13 mg/L (Huang, Yang et al., 2023)). These differences could be ascribed to differences in the enrolled population (most previous studies performed in Asian populations) or in the definition of thrombocytopenia (variable threshold values for numbers of platelets or their percentage of reduction). Other published risk factors include platelet counts <100 to 200 10^9^/L (Chen, Guo et al., 2012; Choi, Lee et al., 2019; Kaya Kılıç, Bulut et al., 2019; Cazavet, Bounes et al., 2020; Lima, Brito et al., 2020; Dai, Jiang et al., 2021) and hepatic disorders (Liao, Dong et al., 2023). They were not detected here, probably because we excluded patients with low basal platelet counts, and our cohort did not include patients with severe hepatic insufficiency. Nevertheless, we noticed elevated C_min_ in 2 patients with mild renal insufficiency associated with liver disease. Our previous retrospective study also identified Charlson comorbidity index ≥4 as a potential risk factor (Thirot, Briquet et al., 2021). This could not be confirmed in this prospective study, possibly because the majority of our patients had fewer comorbidities (11% of patients with an index >4 vs. 22% in the retrospective study) and were not critically-ill (9.5% vs. 37% on ICU).

Notably, our study was the first to evaluate the performance of two predictive scores in identifying the at-risk patients as well as to assess the causality link between linezolid administration and ADRs using the Naranjo scale (Naranjo, Busto et al., 1981), in the line of our previous retrospective study (Thirot, Briquet et al., 2021). We selected this score rather than the WHO-UMC scale (WHO, 2013), because the latter is subject to variations in causality assessment of ADR because of differences in the knowledge and expertise of clinicians who use it, while the Naranjo scale has been designed to reduce inter-rater and intra-rater dissimilarity and is recommended for use in clinical practice and clinical trials (Shukla, Jhaj et al., 2021). Concerning the scores specifically developed to assess linezolid risk of ADR, the Gonzalez-Del Castillo score (Gonzalez-Del Castillo, Candel et al., 2017), which includes platelet levels <90 10^9^/L and hepatic and cerebrovascular disorders as parameters, performed poorly in our study population, again possibly because we excluded patients with hematological disorders at the baseline (platelet levels <75 10^9^/L) and our patients had relatively few co-morbidities. In contrast, the Buzelé score (Buzele, Lemaignen et al., 2015), which combines an age-adjusted Charlson comorbidity index with treatment duration, showed 77% specificity and 67% sensitivity. Thus, in less severely-ill patients with relatively few co-morbidities, as is the case for our study population, the Buzelé score may outperform the Gonzalez-Del Castillo score.

Once patients at risk of developing ADRs have been identified based on these scores, TDM can help to guide dosing to reduce this risk. Based on the 2–8 mg/L recommended therapeutic range for linezolid trough levels (Matsumoto, Shigemi et al., 2014; Fang, Chen et al., 2020; Lin, Hu et al., 2022), 58.7% of our patients experienced supratherapeutic drug exposure, which is similar or higher than reported in earlier prospective [51.4% (Cojutti, Merelli et al., 2019)] or retrospective [33% (Pea, Cojutti et al., 2017)] studies. While the European SmPC and the US label do not recommend dosing adaptation in case of renal insufficiency in spite of reported prolonged half-life in these patients (Zyvox SmPC, 2018; Zyvox US prescribing information 2021), recent works, primarily focusing on critically-ill patients, propose proactive TDM-guided dose adaptation in these populations (Wang, Wang et al., 2021; Wu, Zhang et al., 2022; Shi, Zhang et al., 2023). Our study brings some additional support for such a recommendation also in non-critically-ill patients, with patients with GFR < 60 mL/min/1.73 m^2^ being at particular risk of overdosing.

In our cohort, most of the patients with elevated linezolid trough concentrations developed thrombocytopenia, but others did not, likely due to shorter treatment duration (median of 28 days vs. 8 days for overdosed patients developing thrombocytopenia vs. those who showed no toxicity). Conversely, a minority of patients (*n* = 5) were able to complete >28 days of linezolid treatment without the onset of thrombocytopenia, but their linezolid trough levels remained within the therapeutic range. Our data therefore confirm that proactive TDM could be useful to reduce the risk of thrombocytopenia for long-term treatments (Pea, Viale et al., 2012; Cojutti, Merelli et al., 2019; Lau, Marriott et al., 2023). Although proactive TDM was not part of the design of this study, linezolid dose was reduced in 5 patients with troughs >8 mg/L, but this did not suffice to prevent thrombocytopenia in 2 of them, possibly because TDM and subsequent dose adaptation should come earlier in patient’s management [day 3–5 (Cojutti, Merelli et al., 2019)]. We also noticed in a subset of patients that platelets counts were stable at day 7 (day of the first TDM sample) but decreased during the next week, especially in overdosed patients, which further confirms the need for earlier TDM and dose adaptation to prevent the occurrence of this ADR. TDM could be done as soon as the steady-state is reached in hemodynamically-stable patients, i.e., after 2 days for this short-half life (6h) drug (Fang, Zhang et al., 2021).

Of note, one of the patients with long treatment duration developed peripheral neurotoxicity in spite of adequate trough value, a known ADR of linezolid during long treatment durations as observed in tuberculosis therapy, but which is not considered as related to high C_min_ values (Jaspard, Butel et al., 2020).

Importantly also, we noticed that all 6 ICU patients included in this study had rather low C_min_ values despite a mild to moderate renal insufficiency (median GFR: 53 mL/min/1.73 m^2^; range 32–98 mL/min/1.73 m^2^). Pharmacokinetic variability is well known in critically-ill patients and relies on multiple reasons. Our data highlight a risk of under dosing for these patients who are also at higher risk of severe infections.

Overall, our results strongly support the need of TDM to guide dosing and reduce the risk for developing thrombocytopenia. A recent study in critically-ill patients used model-informed precision dosing (MIPD) to maintain linezolid C_min_ and AUC/MIC within the target range and recommend this practice for patients with renal impairment (Shi, Zhang et al., 2023). Importantly, a strong correlation between C_min_ and AUC values has been demonstrated (Pea, Furlanut et al., 2010; Wu, Zhang et al., 2022), which means in principle that (a) any attempt at maintaining AUC in the therapeutic range will affect C_min_ in parallel and (b) dose adjustment can be performed based on C_min_ regarding both toxicity and efficacy targets, which is easier to perform in clinical practice. However, it is worth mentioning that this correlation is based on population PK models, mostly fed with trough values, and may not be applicable for patients with extreme clearances or if modifying the dosing interval.

Nevertheless, routine TDM can be technically and logistically challenging in clinical practice, especially in outpatients, due to the need to provide regular blood samples taken at exact times (i.e. 12 h for 600 mg linezolid twice daily). We saw indeed large fluctuations in C_min_ values (see Supplementary Figure S1), including in outpatients who were supposed to be more stable over time than ICU patients. It is worth noting that 11/18 cases of thrombocytopenia occurred in outpatients. We show here that samples collected at wrong sampling times can still be clinically relevant by correcting of the sampling time and re-estimating of the “true” trough value using pop-PK model-based simulations. Incorporating such PK models into user-friendly dosing software packages available in clinical hospitals can thus help reduce the burden of TDM on the nursing staff by eliminating the need for blood sampling at exact sampling times.

Our study has some limitations. Although considered sufficient based on our statistical power estimation, the number of patients that could be included over the pre-established recruitment period remains limited. This is due to stewardship policies that do not position linezolid as first-line drug when active and safe alternatives are available. Consequently, only a small number of patients were critically-ill or underwent long-term treatment in our cohort, which restricts the generalizability of our findings in these specific subgroups. On the other hand, the study protocol did not foresee any change in the medical practice, allowing us to capture the reality of routine clinical work and the associated challenges. For instance, blood samples could not be collected as frequently as initially planned, particularly for outpatients who returned to the hospital only for medical consultations.

In conclusion, this medium-sized (*n* = 63) prospective study confirms that treatment duration and supratherapeutic trough values (related to impaired renal function) are independent risk factors for the development of linezolid-induced thrombocytopenia. But this study also brings novel pieces of information. It highlights the interest of Buzelé predictive score in detecting patients at risk of toxicity in non-critically-ill populations and demonstrates the usefulness of the Naranjo score to establish the causal relationship between the observed adverse reaction and the use of linezolid. We also provide evidence that treatment durations could be possibly extended beyond the recommended 28 days as long as drug concentrations are maintained within the therapeutic window, but the risk of neurotoxicity remains a concern in these conditions, as it is not associated with elevated trough concentrations. Patients with mild to moderate renal insufficiency, including when caused by exposure to nephrotoxic drugs, are of particular concern and should benefit from early TDM. Conversely, in critically-ill patients, TDM is important to ensure adequate exposure in order to maximize the chance of therapeutic efficacy while avoiding excessive risk of toxicity.

Our work therefore pleads for the establishment of rules for dose adjustment in patients with renal insufficiency and/or for a systematic use of TDM in patients at-risk, including in non-critically-ill populations. Furthermore, we illustrate how pop-PK models can help to estimate “true” trough levels based on blood samples collected at varying sampling times, reducing the logistical burden of TDM in clinical practice.

## Data Availability

The raw data supporting the conclusion of this article will be made available by the authors, without undue reservation.
