# Supplementary material for: Towards a better detection of patients at-risk of linezolid toxicity in clinical practice: a prospective study in three Belgian hospital centers
Source: Front Pharmacol. 2024 Jan 19;15:1310309. doi: 10.3389/fphar.2024.1310309 (PMC10834751; doi:10.3389/fphar.2024.1310309)
Supplement: Supplementary file 1 [file DataSheet1.PDF]

Towards a better detection of patients at-risk of linezolid toxicity in clinical practice: a prospective study in three Belgian hospital centers

Hélène Thirot,[1,2] David Fage,[3] Antonia Leonhardt,[4] Philippe Clevenbergh,[5] Tatiana Besse,[5] Jean Cyr Yombi,[6] Olivier Cornu,[6] Caroline Briquet,[6] Maya Hites,[7] Frédérique Jacobs,[7] Gert-Jan Wijnant,[1] Sebastian G. Wicha,[4] Frédéric Cotton,[3] Paul M. Tulkens,[1] Anne Spinewine,[2] Françoise Van Bambeke.[1]

[1] Pharmacologie cellulaire et moléculaire, Louvain Drug Research Institute, Université catholique de Louvain, Brussels, Belgium ; [2] Clinical Pharmacy, Louvain Drug Research Institute, Université catholique de Louvain, Brussels, Belgium ; [3] Department of Clinical Chemistry, Laboratoire Hospitalier Universitaire de Bruxelles (LHUB-ULB), Brussels, Belgium ; [4] Department of Clinical Pharmacy, Institute of Pharmacy, University of Hamburg, Hambourg, Germany ; [5] University Hospital Brugmann, Université Libre de Bruxelles, Brussels, Belgium ; [6] Cliniques Universitaires Saint-Luc, Université catholique de Louvain, Brussels, Belgium ; [7] Hôpitaux Universitaires de Bruxelles-Erasme (HUB), Université Libre de Bruxelles, Brussels, Belgium.

SUPPLEMENTARY MATERIAL

Methods and results S1: Pharmacokinetic modelling of linezolid trough levels

The standard dosing of linezolid is 600 mg bid. The aimed for trough values are 2-8 mg/L. In reality, not all trough samples are drawn at 12 h, especially in non-hospitalized patients. For better comparability of the subject's trough values, the concentrations at 12 h after dose were simulated based on the actually measured values.

Published models for linezolid in infected patients were evaluated. Only models with oral administration were considered, and those using covariates that were not assessed in our patients were excluded. This left 5 different models (Table A):

Table A: Considered models with study population, base model, and covariates. GPC: Gram-positive cocci, MRSA: Methicillin-resistant *Staphylococcus aureus,* CMT: Compartments, CL: Clearance, V: Volume of distribution, CLCR: Creatinine Clearance, TBW: Total body weight, MM-elimination: Michaelis-Menten-elimination.

	Population	Base model	Covariates
**Matsumoto, 2014 (1)**	Adult Japanese patients	1 CMT, 1st order absorption, 1st order elimination	CLCR on CL
**Abe, 2009 (2)**	Adult Japanese/ Caucasian patients	1 CMT, 1st order absorption, 1st order elimination	TBW on CL, V<br>Age on CL
**Tsuji, 2017 (3)**	Adult Japanese patients w. GPC or MRSA infections	2 CMT, 1st order absorption, 1st order elimination	TBW on CL<br>Age on CL
**Boak, 2014 (4)**	Adult patients (Australia, USA)	1 CMT, 3 absorption lag CMT, 1st order elimination	CLCR on CL<br>TBW on CL
**Plock, 2007 (5)**	Healthy volunteers and septic patients	2 CMT, linear absorption, MM-elimination & linear elimination	-

Model predictions of the five models presented in Table A were calculated for two time points: At the time of the actual sampling, to show the fit of the model to the actually measured values, and at 12 h, for better between subject comparability (Tables B and C).

Although some of the samples were taken from the same patient, every dosing event was treated individually due to the time difference between the samples.

The different models' predictions were compared by Bland-Altman-Plots to evaluate the agreement of the models (Figures A-J).

For the final 12 h trough concentrations, ultimately the model from Plock et al. (ref) was selected. The model from Plock was chosen because the population studied by Plock et al. is the most equivalent to our patients, and it also showed the best fit.

The 12 h troughs can differ from the measured trough values, especially for the samples that are taken well before 12 h after dose. As a consequence, dose adjustments might be necessary to reach the targeted trough concentration of 2-8 mg/L.

Table B: Observed Linezolid (LZD) concentrations vs. model-predicted values for each utilized model.

Patient	Dosing [hhmm]	Sampling [hhmm]	Delta [hhmm]	Observed LZD [mg/L]	Matsumoto [mg/L]	Abe [mg/L]	Tsuji [mg/L]	Boak [mg/L]	Plock [mg/L]
**A.1.1**	7h00	14h30	7h30	**13.70**	11.83	13.31	10.77	11.54	13.57
**A.2.1**	9h00	15h00	6h00	**8.20**	8.05	8.09	6.35	7.36	8.15
**A.2.2**	9h45	16h00	6h15	**5.50**	5.87	5.51	5.77	5.29	5.53
**A.2.3**	9h30	14h45	5h15	**6.30**	7.41	6.31	6.57	6.09	6.36
**A.3.1**	9h00	16h10	7h10	**16.30**	13.48	15.77	12.22	13.42	16.14
**A.5.1**	7h00	17h05	10h	**8.00**	7.06	7.89	7.05	7.11	7.93
**A.7.1**	7h30	12h15	5h15	**21.30**	18.44	20.51	16.33	17.40	21.09
**A.7.2**	18h00	12h30	18h	**7.30**	6.54	7.21	6.75	6.49	7.23
**A.9.1**	17h00	10h30	17h	**14.1**	12.12	13.61	11.21	11.81	13.97
**A.9.2**	17h00	10h30	17h	**12.3**	10.44	11.91	9.79	10.38	12.19
**A.10.1**	14h20	8h00	6h00	**23.10**	19.21	22.45	18.94	18.99	22.87
**A.10.2**	15h20	9h00	6h00	**22.10**	18.49	21.51	18.26	18.24	21.88
**A.10.3**	9h15	14h50	5h35	**28.40**	22.70	27.43	22.08	22.83	28.12
**A.10.2.1**	7h10	16h10	9h00	**27.40**	21.66	26.50	21.41	22.18	27.13
**A.14.1**	8h30	11h30	3h00	**9.80**	12.07	9.79	9.43	9.88	10.02
**A.17.1**	20h00	11h30	15h30	**8.00**	7.27	7.89	7.88	7.17	7.92

Table C: Predicted trough concentrations at 12 h.

Patient	Matsumoto [mg/L]	Abe [mg/L]	Tsuji [mg/L]	Boak [mg/L]	Plock [mg/L]
**A.1.1**	6.44	11.05	9.27	9.32	8.05
**A.2.1**	2.11	5.17	5.21	4.51	2.64
**A.2.2**	1.21	2.68	3.89	2.54	1.42
**A.2.3**	1.21	2.87	4.45	2.62	1.20
**A.3.1**	7.38	13.33	10.60	11.03	10.36
**A.5.1**	5.17	6.86	6.33	6.14	6.18
**A.7.1**	9.01	16.48	13.54	13.34	12.00
**A.7.2**	11.72	8.49	9.02	9.00	11.93
**A.9.1**	17.50	13.98	12.54	13.72	18.90
**A.9.2**	15.56	12.29	11.05	12.26	16.91
**A.10.1**	10.75	18.05	15.98	14.51	14.83
**A.10.2**	10.10	17.12	15.32	13.77	13.91
**A.10.3**	13.33	22.66	18.82	17.94	19.11
**A.10.2.1**	17.44	24.36	19.88	20.00	23.23
**A.14.1**	1.83	4.65	6.00	2.25	1.19
**A.17.1**	10.48	9.75	11.93	8.93	10.82

Bland-Altman-Plots show the level of agreement between the estimated values of the different models.

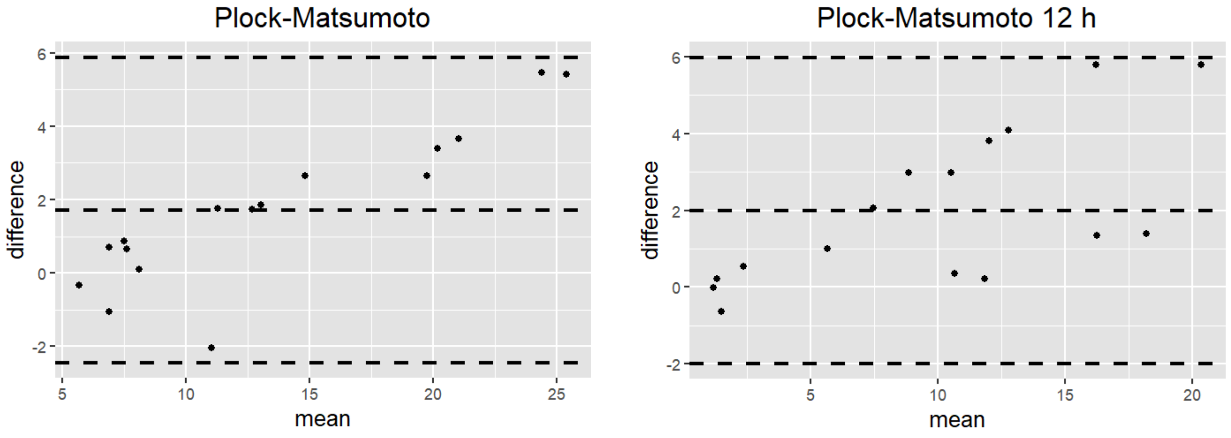

Figure A: Bland-Altman Plots for comparison between models from Plock and Matsumoto

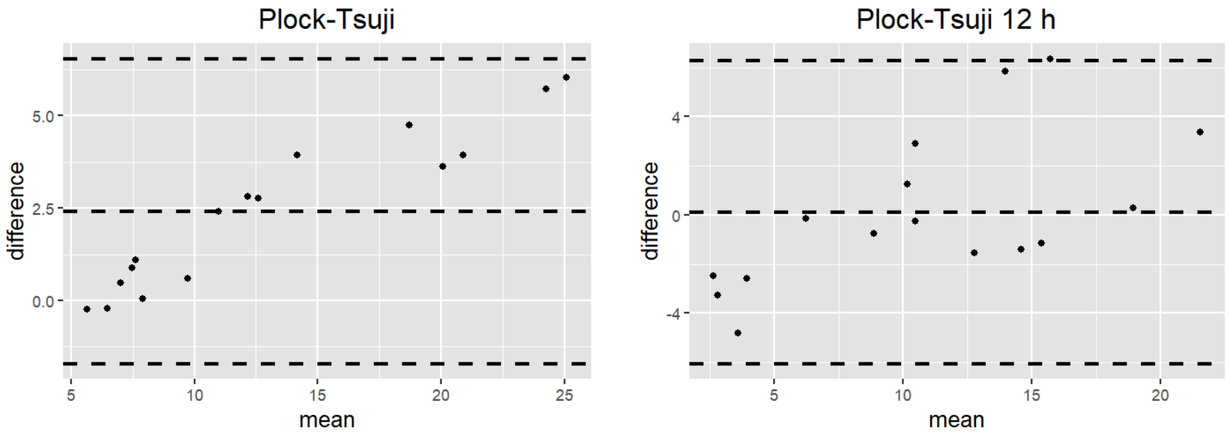

Figure B: Bland-Altman Plots for comparison between models from Plock and Tsuji

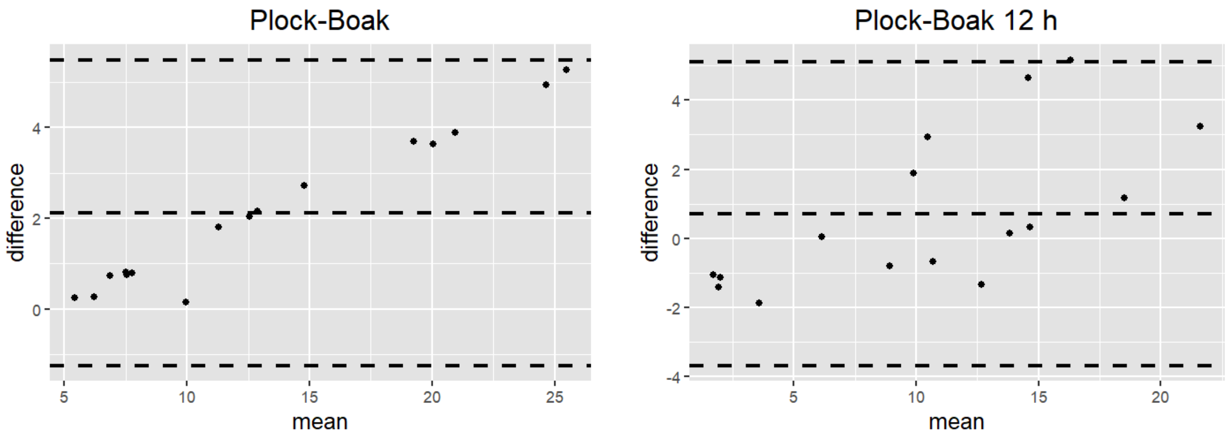

Figure C: Bland-Altman Plots for comparison between models from Plock and Boak

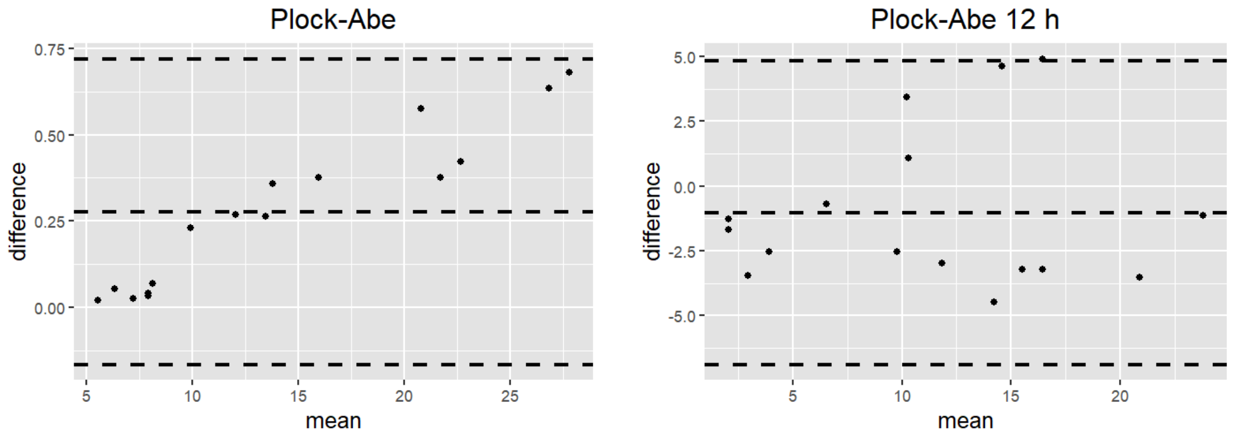

Figure D Bland-Altman Plots for comparison between models from Plock and Abe

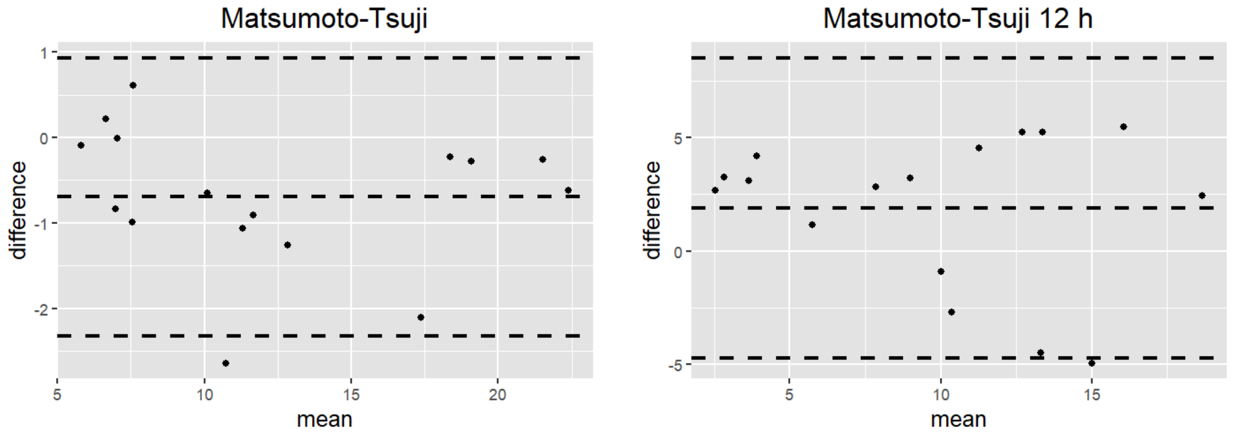

Figure E: Bland-Altman Plots for comparison between models from Matsumoto and Tsuji

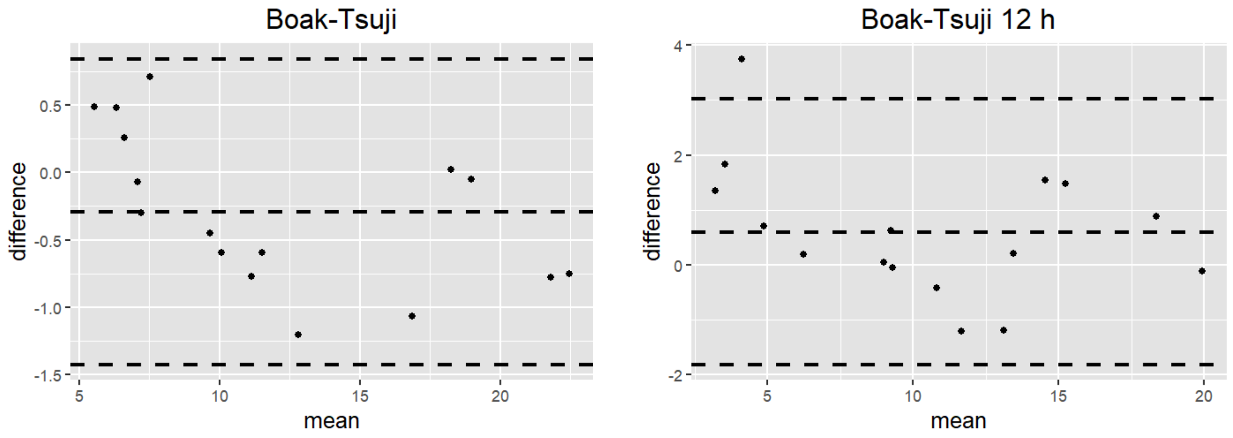

Figure F: Bland-Altman Plots for comparison between models from Boak and Tsuji

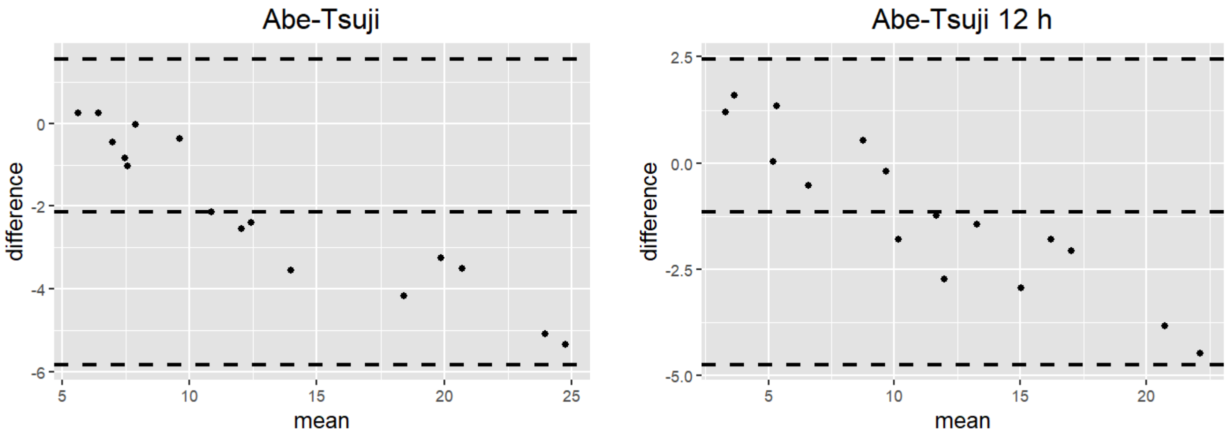

Figure G: Bland-Altman Plots for comparison between models from Abe and Tsuji

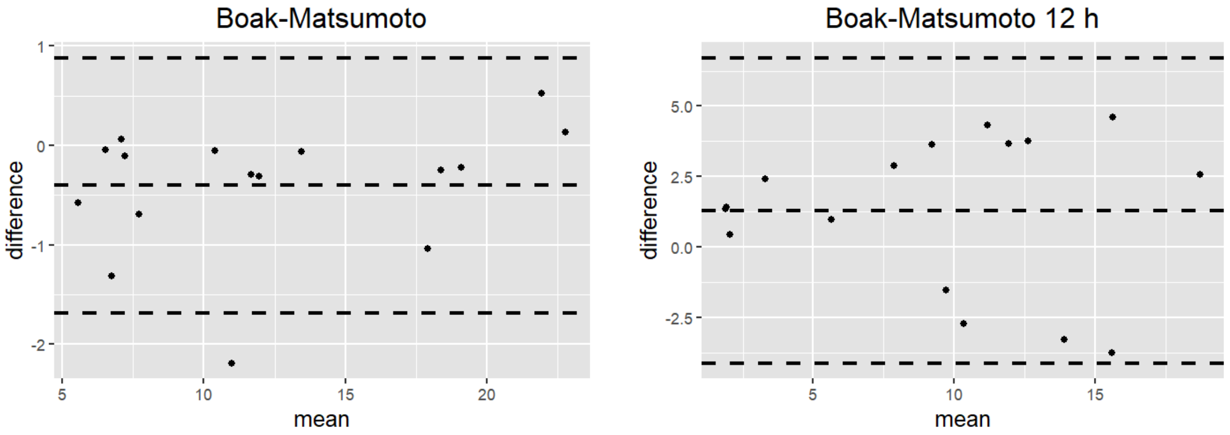

Figure H: Bland-Altman Plots for comparison between models from Boak and Matsumoto

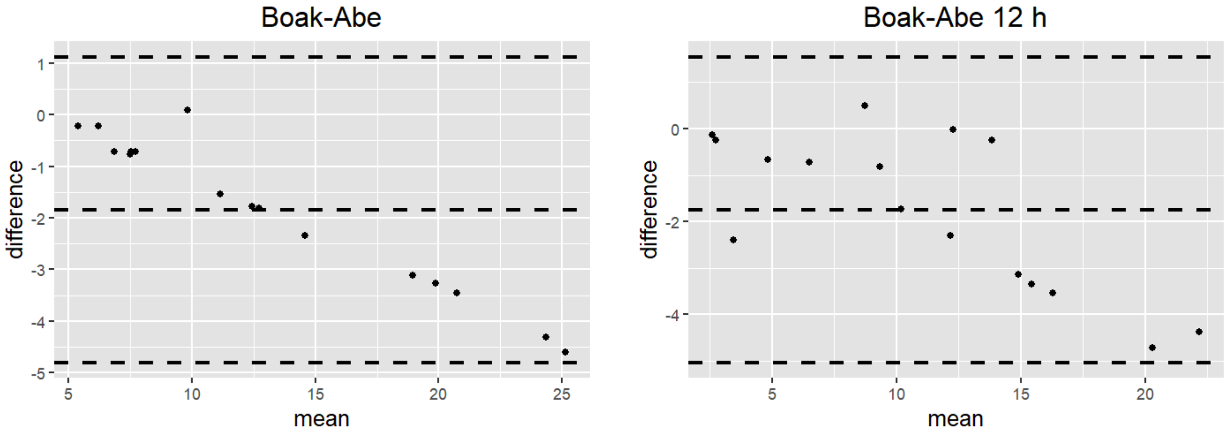

Figure I: Bland-Altman Plots for comparison between models from Boak and Abe

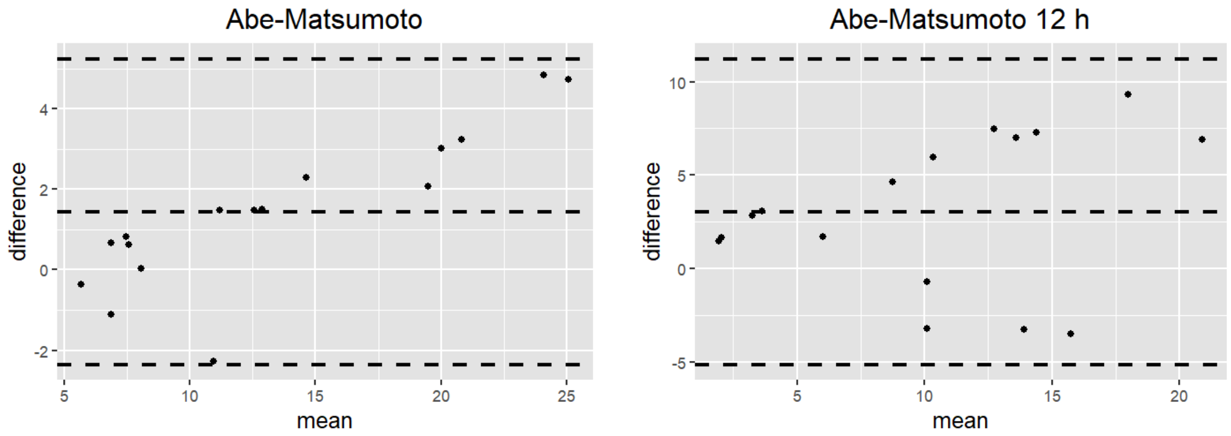

Figure J: Bland-Altman Plots for comparison between models from Abe and Matsumoto

Table S1: calculation of the sample size.

The sample size was calculated based on the study of Dong et al (6). These authors found that patients with thrombocytopenia had significantly higher Cmin (8.81 mg/L than those who did not develop this ADR (C_{min}: 2.88 mg/L [0.35–8.78]). On this basis, a sample size of 20 patients developing thrombocytopenia and 20 who do not develop thrombocytopenia allows to achieve 95% power to reject the null hypothesis of equal linezolid blood concentration when the population blood concentration difference is µ1 - µ2 = 5.9, with a standard deviation for both groups of 5 and with a significance level (alpha) of 0.05 using a two-sided two-sample equal-variance t-test. In our retrospective study (7), the thrombocytopenia frequency was 19%. Therefore, to reach the minimal number of 20 patients who will develop thrombocytopenia, we should enroll 105 patients.

Two-Sample T-Tests Assuming Equal Variance

Numeric Results for Two-Sample T-Test Assuming Equal Variance
Alternative Hypothesis: µ1 ≠ µ2 where µ1 and µ2 are the Cmin values of 8.81 and 2.88 mg/L reported in the Dong et al. study

Target Power	Actual Power	N1	N2	N	µ1	µ2	µ1 - µ2	σ	Alpha
0.80	0.93401	4	4	8	8.8	2.9	5,9	2.0	0.050
0.85	0.93401	4	4	8	8.8	2.9	5.9	2.0	0.050
0.95	0.98279	5	5	10	8.8	2.9	5.9	2.0	0.050
0.80	0.82661	13	13	26	8.8	2.9	5.9	5.0	0.050
0.85	0.85545	14	14	28	8.8	2.9	5.9	5.0	0.050
0.95	0.95488	20	20	40	8.8	2.9	5.9	5.0	0.050
0.80	0.80331	46	46	92	8.8	2.9	5.9	10.0	0.050
0.85	0.85642	53	53	106	8.8	2.9	5.9	10.0	0.050
0.95	0.95030	75	75	150	8.8	2.9	5.9	10.0	0.050

References

- Julious, S. A. 2010. Sample Sizes for Clinical Trials. Chapman & Hall/CRC. Boca Raton, FL.
- Chow, S.-C., Shao, J., and Wang, H. 2008. Sample Size Calculations in Clinical Research (Second Edition). Chapman & Hall/CRC. Boca Raton, FL.
- Machin, D., Campbell, M., Fayers, P., and Pinol, A. 1997. Sample Size Tables for Clinical Studies, 2nd Edition. Blackwell Science. Malden, MA.
- Zar, Jerrold H. 1984. Biostatistical Analysis (Second Edition). Prentice-Hall. Englewood Cliffs, New Jersey.

Report Definitions
Target Power is the desired power value (or values) entered in the procedure. Power is the probability of rejecting a false null hypothesis.
Actual Power is the power obtained in this scenario. Because N1 and N2 are discrete, this value is often (slightly) larger than the target power.
N1 and N2 are the number of items sampled from each population.
N is the total sample size, N1 + N2.
µ1 and µ2 are the assumed population means for power and sample size calculations.
µ1 - µ2 is the difference between population means at which power and sample size calculations are made.
σ is the assumed population standard deviation for each of the two groups.
Alpha is the probability of rejecting a true null hypothesis.

Summary Statements
Group sample sizes of 20 and 20 achieve 95% power to reject the null hypothesis of equal means when the population mean difference is µ1 - µ2 = 8.8 – 2.9 = 5.9 with a standard deviation for both groups of 5.0 and with a significance level (alpha) of 0.050 using a two-sided two-sample equal-variance t-test.

Table S2: subpopulation analysis of patient groups that may be at risk for under- or overexposure to linezolid

A. ***Obese patients***: (BMI > 30 kg/m^2 = 24 patients; mean BMI = 36.35 kg/m^2 (30.1 – 46)): no trend to underdosing

**Parameters**	**Median (range) or N**
Age (years)	62.5 (31-83)
Male/Female	18/6
GFR (mL/min/1.73 m^2)	78.5 (10-134)
DPKD (mg/kg/day)[a]	10.9 (7.8-14.4)
Charlson score	1.5 (0-6)
Outpatients/inpatients	11/13
Treatment duration (days)	21.5 (5-121)
Patients developing ADR	17
Stop for toxicity	4
Thrombocytopenia	8
LZD C_{min} (AVG) (mg/L)	8.3 (0-25.4)

[a] Daily Per Kilogram Dose

B. ***High renal clearance*** (GFR > mL/min/1.73 m^2: 14 patients; Mean GFR = 115 (103-134) mL/min/1.73 m^2): trend to lower C_{min}

**Parameters**	**Median (range) or N**
Age (years)	46.79 (31-64)
Male/Female	6/8
Weight (kg)	76 (57-118)
DPKD (mg/kg/day)	15.8 (10.2-21.2)
Charlson score	1 (0-3)
Outpatients/inpatients	4/10
Treatment duration (days)	12.5 (5-85)
Patients developing ADR	4
Stop for toxicity	0
Thrombocytopenia	1
LZD C_{min} (AVG) (mg/L)	3.82 (1.3-13.4)

	**GFR <** mL/min/1.73 m^2	**GFR >** mL/min/1.73 m^2	**p-value**
**LZD C_{min} (mg/L)**	10.82 (0-40.85)	3.82 (1.3-13.4)	0.003

C. ICU patients (N =6) : Trend to low C_{min}

**Parameters**	**Median (range) or N**
Age (years)	69.5 (64-97)
Male/Female	6/0
GFR (mL/min/1.73 m^2)	53 (32-98)
Weight (kg)	81 (63-116)
DPKD (mg/Kg/day)	15 (10.3-19)
Charlson	1.5 (0-6)
Outpatients/inpatients	0/6
Treatment duration (days)	8.5 (7-17)
Patients developing ADR	3
Stop for toxicity	0
Thrombocytopenia	0
LZD C_{min} (AVG) (mg/L)	3 (0-5.7)

D. Patients with liver disorder (N=8; 7 steatosis and 1 cirrhosis, based on patient's files): trend to high C_{min}

**Parameters**	**Median (range) or N**
Age (years)	62.5 (43-89)
Male/Female	6/2
GFR (mL/min/1.73 m^2)	68.5 (38-110)
Weight (kg)	89.25 (61-104)
DPKD (mg/kg/day)	13.4 (11.5-19.7)
Charlson	3.5 (2-6)
Outpatients/inpatients	1/7
Treatment duration (days)	13 (4-17)
Patients developing ADR	3
Stop for toxicity	1
Thrombocytopenia	2
LZD C_{min} (AVG) (mg/L)	8.7 (0-40.85)

Table S3: Number of serotonergic agents prescribed

Serotonergic Agents	N drugs
SSRI	8
TCA	3
Mirtazapine/trazodone	10
Tramadol	19
Triptan	1

Table S4: Individual Gonzalez score for patients developing hematological toxicity

Patients	Basal platelet count < 90 10^9/L	Renal failure [a]	Moderate or severe liver disease	Cerebrovascular disease	Total
A.3	0	0	0	0	0
A.4	0	2	0	0	2
B.3	0	2	0	0	2
A.6	0	0	0	0	0
A.7	0	0	0	0	0
A.8	0	2	0	0	2
B.5	0	0	0	2	2
B.10	0	2	0	0	2
A.9	0	2	0	0	2
A.10.1	0	0	0	0	0
A.10.2	0	0	0	0	0
B.15	0	2	0	2	4
B.19	0	2	2	0	4
A.15	0	2	0	0	2
A.17	0	0	0	0	0
B.23	0	2	0	0	2
B.24	0	0	0	2	2
B.26	0	0	0	0	0
D.4	0	2	0	0	2
D.5	0	0	2	0	2

[a] Creatinine clearance < 50mL/min

Table S5: Individual Buzelé 's score for patients developing ADRs

Patients	Charlson Comorbidity index [a]	Each decade of age above 40	Each complete week of linezolid therapy	Total
A.1	0	2	3	5
A.2	1	1	15	17
A.3	0	2	5	7
A.4	0	3	4	7
A.5	1	1	17	19
A.6	0	1	3	4
A.7	1	2	7	10
B.4	5	1	2	8
A.8	1	3	4	8
B.5	3	2	2	7
B.10	0	3	1	4
A.9	4	2	6	12
A.10.1	2	3	4	9
B.12	1	0	1	2
B.15	5	2	1	8
B.17	6	2	2	10
B.19	2	0	2	4
B.21	3	4	0	7
B.22	2	4	2	8
A.10.2	2	3	4	9
A.14	0	0	12	12
A.15	5	2	1	8
A.16	6	3	3	12
A.17	0	2	5	7
B.23	1	4	2	7
B.24	2	3	1	6
B.26	1	0	1	2
A.18.1	2	0	3	5
A.18.2	2	0	4	6
D.4	4	3	1	8
D.5	4	2	2	8
B.40	2	2	1	5
B.41	0	4	1	5

[a] Charlson comorbidity index (8)

Table S6: Characteristics of patients with or without thrombocytopenia

Parameters	No thrombocytopenia N = 45 N (%) or median (range)	Thrombocytopenia (n = 18) N (%) or median (range)	P-value
Patients'characteristics			
Male (%)	25 (55.6)	14 (77.8)	0.086
Age (year)	63 (31-97)	68.5 (33-83)	0.212
Weight (kg)	76.1 (40-132)	88.75 (52-154)	0.512
Body mass index (kg/m^2)	26.99 (14.9-46)	29.17 (19.9-41.7)	0.554
Inpatients/Outpatients	37/8	7/11	0.076
Diabetes (%)	15 (33.3)	6 (33.3)	1
Charlson index	2 (0-6)	2 (0-6)	0.822
GFR (mL/min/1.73 m^2)	74 (6-134)	50 (5-115)	***0.026***
Renal insufficiency [a]	14 (31.1)	11 (61.1)	***0.028***
ALT	16 (5-137)	10 (5-162)	0.054
GGT	54.75 (8-992)	56.5 (15-191)	0.883
AST	20 (0.2-119)	17 (0.3-399)	0.335
Bilirubin	0.4 (0.1-9)	0.5 (0.2-31.1)	0.585
Basal platelet count (x10^9/L)	338 (136-843)	253 (130-463)	***0.017***
Antimicrobial treatment			
Treatment duration (days)	10 (4-121)	21 (9-53)	***0.03***
Previous treatment with vancomycin	11 (24.4)	10 (55.6)	***0.018***
First line drug	9 (20)	3 (16.7)	0.533
Route of administration (IV/PO)	8/37	1/17	0.201
Dose/kg/J	15.8 (9.1-30)	13.5 (7.8-22.9)	0.501
Average C_{min} (mg/L)	7.6 (0-34)	15.3 (4.7-40.85)	***0.001***
Comedications			
Heparin	21 (46.7)	8 (44.4)	0.873
Amiodarone	1 (2.2)	2 (11.1)	0.194
Amlodipine	10 (22.2)	4 (22.4)	1
Omeprazole	3 (6.7)	3 (16.7)	0.222
Pyridoxine (vit B6)	10 (22.2)	3 (16.7)	0.453

[a] defined as a GFR < 60 mL/min

Figure S1. Individual values of linezolid C_{min} and basal renal function (basal glomerular filtration rate) for each patient. In the upper panel, black dots refer to concentrations measured for daily doses of 600 mg 2x/day and red dots, for daily doses of 600 mg 1x/day. In the lower panel, arrows point to patients with hepatic disorders. The green zone corresponds to target values of C_{min} (2-8 mg/L; upper panel) or normal renal function (GFR > 90 mL/min/1.73 m^2; lower panel), the red zone, to higher C_{min} (upper panel) or GFR < 60 mL/min/1.73 m^2 (lower panel), the yellow zone in the lower panel, to intermediate GFR (60-90 mL/min/1.73 m^2). Among patients with C_{min} values available (61), 37 had at least 1 sample with C_{min} > 8 mg/L, among them, 20 and 12 patients had a GFR < mL/min/1.73 m^2 and in the 60-90 ml/min interval, respectively.

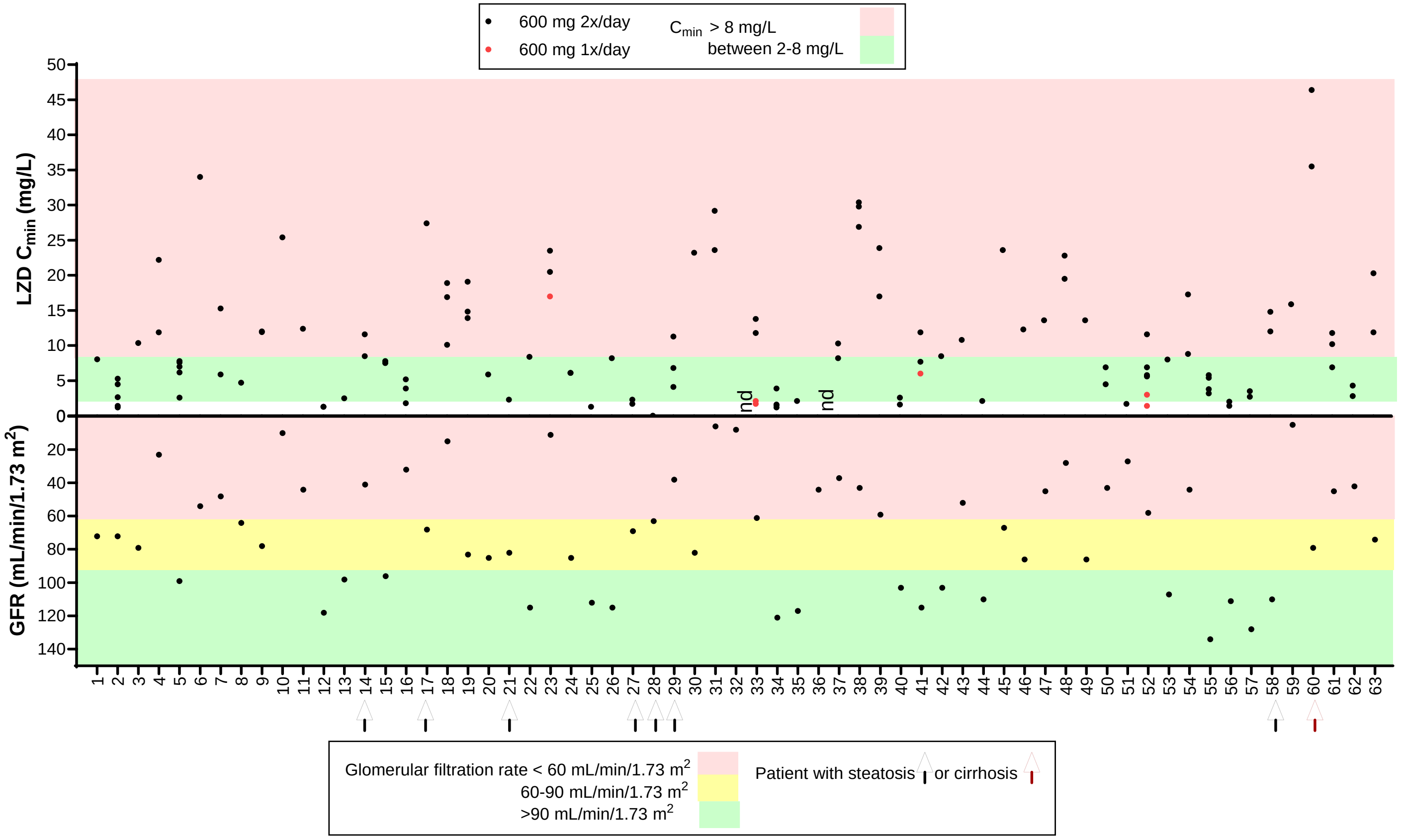

Figure S2: Correlation between individual C_{min} and GFR (measured the day TDM was performed) for each patient (left) or correlation between average C_{min} and the percentage of change in platelet counts (right; difference between counts at the end of the treatment and at day 0 expressed in percentage; a negative value means a reduction). The red dots in the left graph points to patients with a high C_{min} (27 and 46 mg/L) but a GFR at 69 and 89 mL/min/1.73 m^2, respectively. These patients have liver disease (steatosis and cirrhosis, respectively).

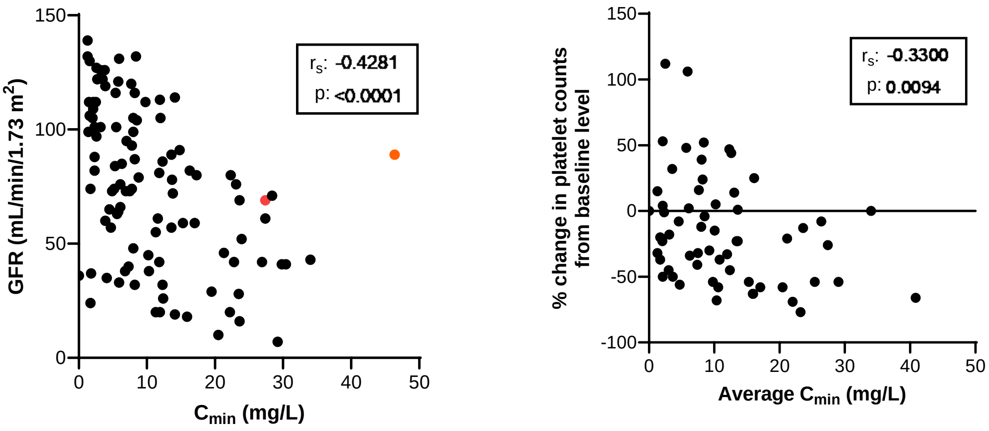

Figure S3. Platelets counts and C_{min} in patients having benefited from dose readjustment based on TDM data. The yellow zone corresponds to thrombocytopenia, the dotted red line, to the maximal target C_{min} (8 mg/L), and the gray arrow point to the day of dosing readjustment (from 600 mg q12h to 600 mg q24h).

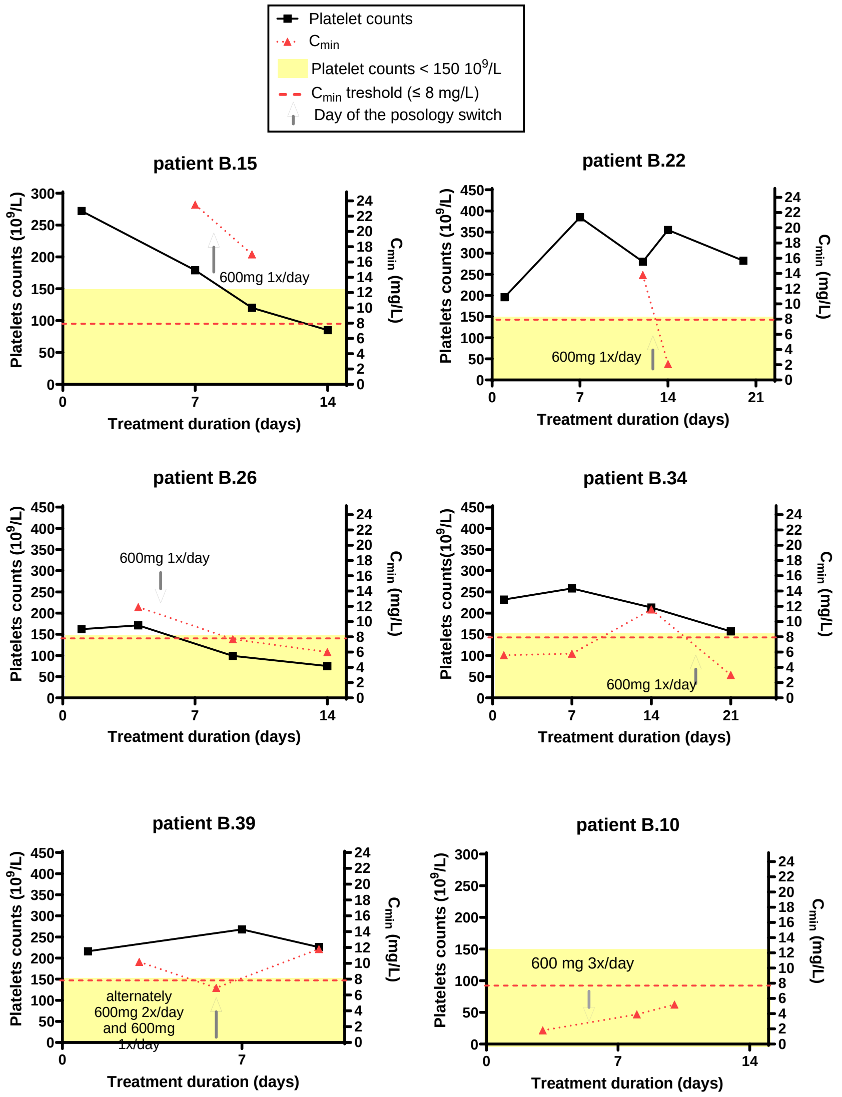

Figure S4. Average C_{min} value in patients developing or not adverse drug reactions. Horizontal lines show the median and interquartile range. The green zone highlights the target C_{min} window (2-8 mg/L). A significant difference is observed only between patients without ADR and patients developing thrombocytopenia (p-value = 0.011).

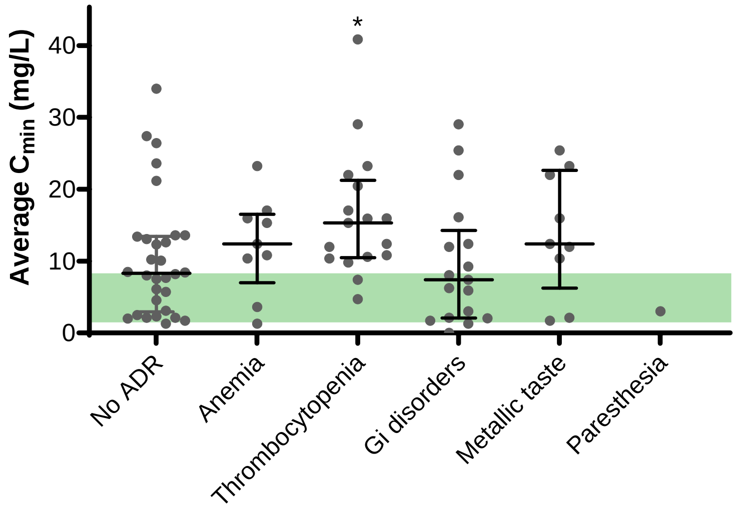

Figure S5. Receiver operating characteristic (ROC) curve of Gonzales-Del Castillo's (left) and Buzele's (right) scores to evaluate the probability of association between ADR (left) or thrombocytopenia (right) and linezolid administration. No cutoff could be established for the Gonzales Del Castillo score. The cut-off value was 7 for Buzele's score, with a specificity of 76.7% and a sensitivity of 66.6%.

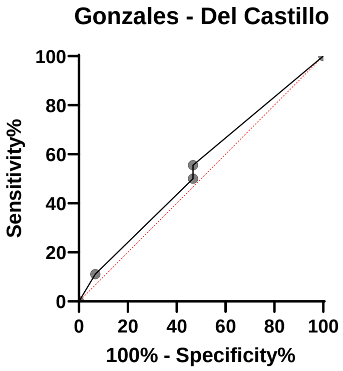

Area	0.5407
Std. Error	0.08105
95% confidence interval	0.3819 to 0.6996
P value	0.6156

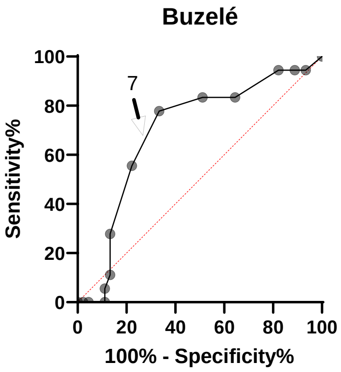

Area	0.6951
Std. Error	0.07402
95% confidence interval	0.5500 to 0.8401
P value	0.0162

Figure S6. Receiver operating characteristic (ROC) curve for four parameters included in the univariate analysis of the risk factors for thrombocytopenia, with determination of the corresponding cut-off values.

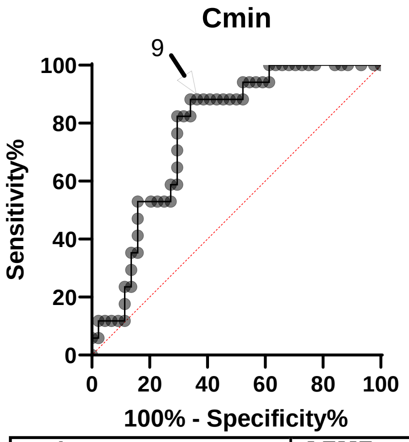

Area	0.7687
Std. Error	0.06047
95% confidence interval	0.6502 to 0.8872
P value	0.0012

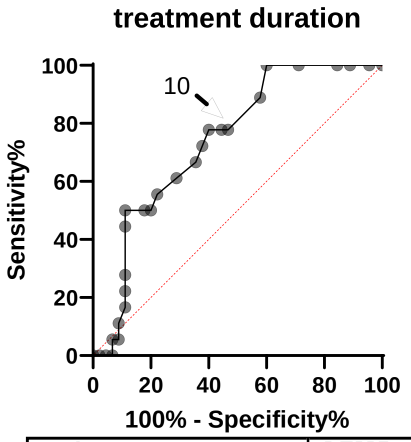

Area	0.7395
Std. Error	0.06358
95% confidence interval	0.6149 to 0.8641
P value	0.0032

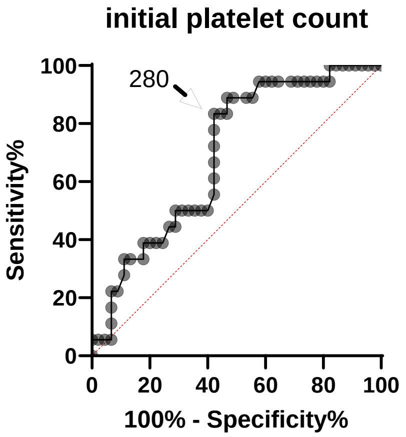

Area	0.6938
Std. Error	0.06868
95% confidence interval	0.5592 to 0.8284
P value	0.0169

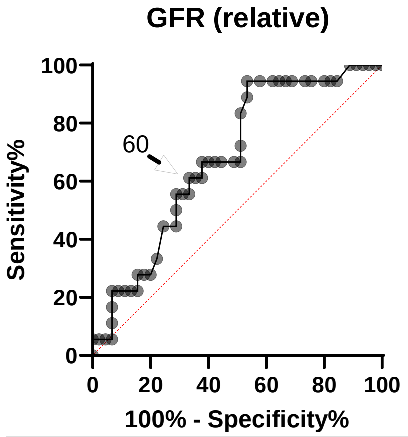

Area	0.6790
Std. Error	0.06991
95% confidence interval	0.5420 to 0.8160
P value	0.0274

Figure S7: Receiver operating characteristic (ROC) curves for GFR to determine cut-off value for higher risk of thrombocytopenia, comparing GFR calculated using the CKD-EPI formula as in Figure S6 or the corresponding absolute value, applying the following formula (9):

$BSA = \sqrt{\frac{Height\ (cm) * weight\ (kg)}{3600}}$ and $GFR\ (mL/min) = GFR\ (mL/min/\ 1.73\ m^2\ ) * BSA/1.73$

The right panel shows the correlation between these two values for each individual patient (3 patients excluded, as their height was not indicated in the patient's files).

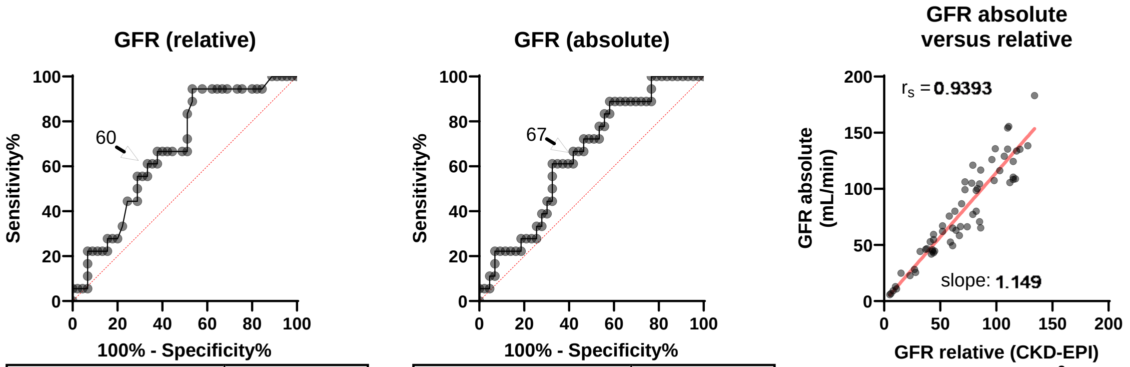

Area	0.6790
Std. Error	0.06991
95% confidence interval	0.5420 to 0.8160
P value	0.0274

Area	0.6512
Std. Error	0.07240
95% confidence interval	0.5093 to 0.7931
P value	0.0643

Figure S8. Evolution of platelet counts over time vs C_{min} values. The yellow zone corresponds to thrombocytopenia and the dotted red line, to the maximal target C_{min} (8 mg/L).

A. Patients with at least 2 dosages (at approx. day 7 and day 14)

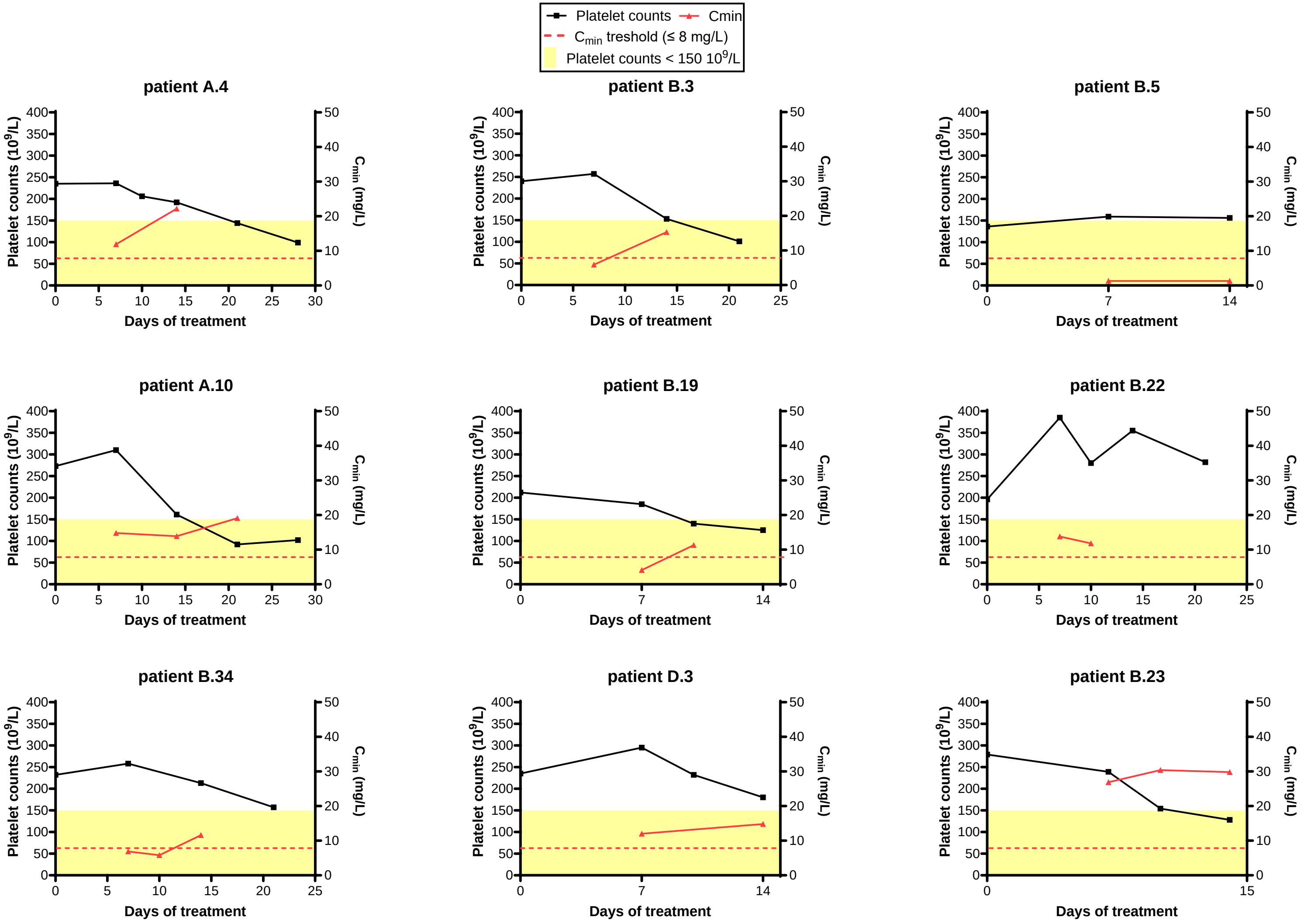

B. Patients developing thrombocytopenia

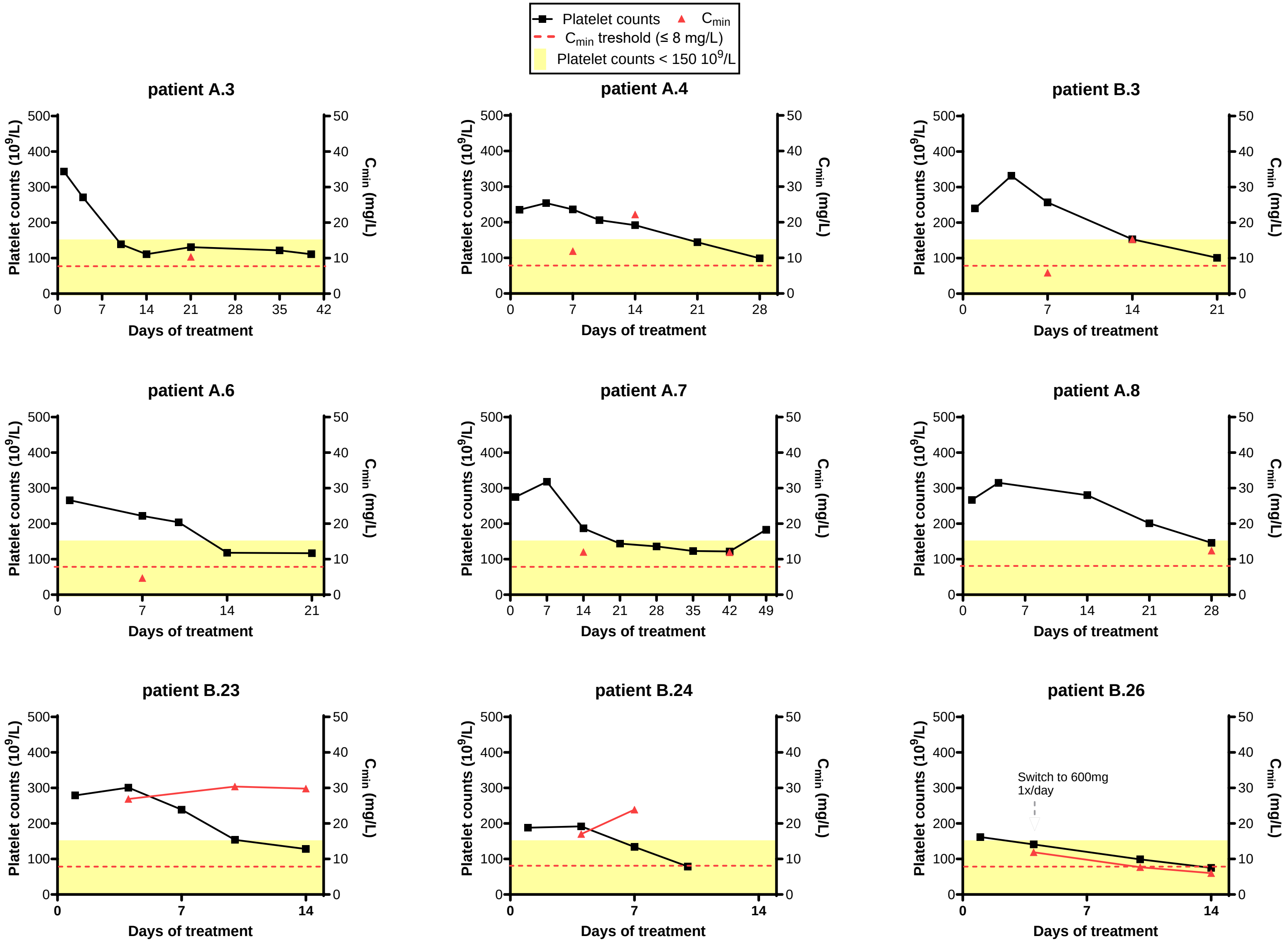

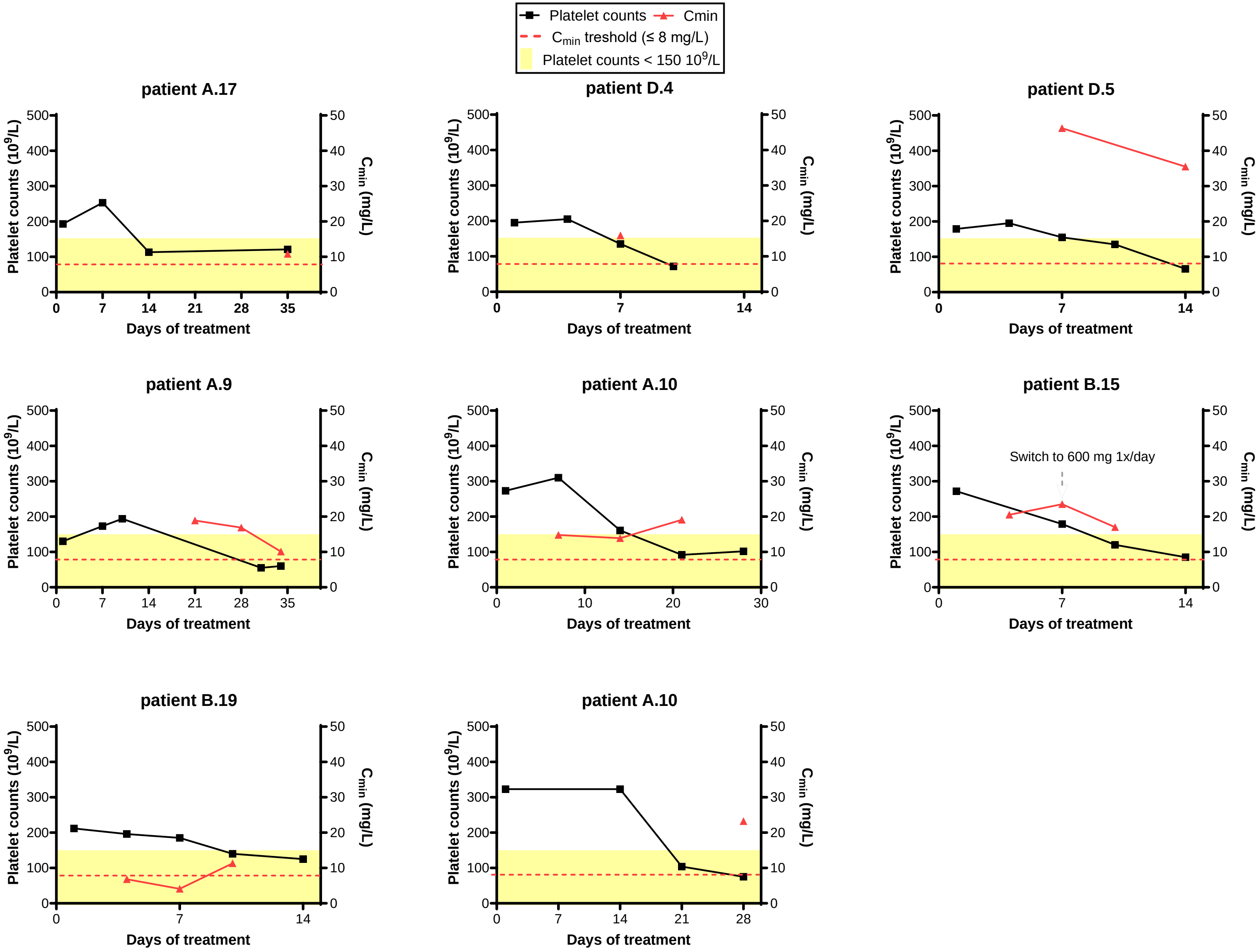
Platelet counts
Cmin
Cmin treshold (≤ 8 mg/L)
Platelet counts < 150 10^9/L
patient A.17
patient D.4
patient D.5
patient A.9
patient A.10
patient B.15
Switch to 600 mg 1x/day
patient B.19
patient A.10
Platelet counts (10^9/L)
Cmin (mg/L)
Days of treatment

Figure S9. Evolution of platelet counts over time vs C_{min} values in patients with treatment duration ≤ 14 days and high C_{min}, including after the interruption of the treatment (gray line). The yellow zone corresponds to thrombocytopenia and the dotted red line, to the maximal target C_{min} (8 mg/L).

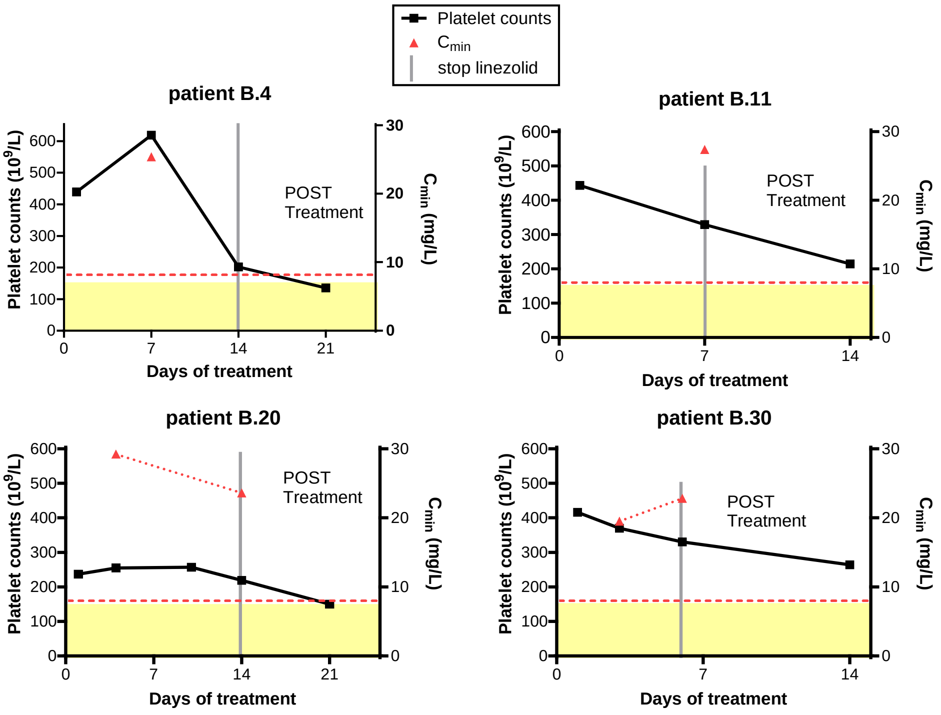

References

1. **Matsumoto K, Shigemi A, Takeshita A, Watanabe E, Yokoyama Y, Ikawa K, Morikawa N, Takeda Y.** 2014. Analysis of thrombocytopenic effects and population pharmacokinetics of linezolid: a dosage strategy according to the trough concentration target and renal function in adult patients. Int J Antimicrob Agents **44:**242-247.
2. **Abe S, Chiba K, Cirincione B, Grasela TH, Ito K, Suwa T.** 2009. Population pharmacokinetic analysis of linezolid in patients with infectious disease: application to lower body weight and elderly patients. J Clin Pharmacol **49:**1071-1078.
3. **Tsuji Y, Holford NHG, Kasai H, Ogami C, Heo YA, Higashi Y, Mizoguchi A, To H, Yamamoto Y.** 2017. Population pharmacokinetics and pharmacodynamics of linezolid-induced thrombocytopenia in hospitalized patients. Br J Clin Pharmacol **83:**1758-1772.
4. **Boak LM, Rayner CR, Grayson ML, Paterson DL, Spelman D, Khumra S, Capitano B, Forrest A, Li J, Nation RL, Bulitta JB.** 2014. Clinical population pharmacokinetics and toxicodynamics of linezolid. Antimicrob Agents Chemother **58:**2334-2343.
5. **Plock N, Buerger C, Joukhadar C, Kljucar S, Kloft C.** 2007. Does linezolid inhibit its own metabolism? Population pharmacokinetics as a tool to explain the observed nonlinearity in both healthy volunteers and septic patients. Drug Metab Dispos **35:**1816-1823.
6. **Dong HY, Xie J, Chen LH, Wang TT, Zhao YR, Dong YL.** 2014. Therapeutic drug monitoring and receiver operating characteristic curve prediction may reduce the development of linezolid-associated thrombocytopenia in critically ill patients. Eur J Clin Microbiol Infect Dis **33:**1029-1035.
7. **Thirot H, Briquet C, Frippiat F, Jacobs F, Holemans X, Henrard S, Tulkens PM, Spinewine A, Van Bambeke F.** 2021. Clinical Use and Adverse Drug Reactions of Linezolid: A Retrospective Study in Four Belgian Hospital Centers. Antibiotics (Basel) **10:**530.
8. **Charlson ME, Pompei P, Ales KL, MacKenzie CR.** 1987. A new method of classifying prognostic comorbidity in longitudinal studies: development and validation. J Chronic Dis **40:**373-383.
9. **Redal-Baigorri B, Rasmussen K, Heaf JG.** 2013. The use of absolute values improves performance of estimation formulae: a retrospective cross sectional study. BMC Nephrol **14:**271.
